# Alignment of 1000 Genomes Project reads to reference assembly GRCh38

**DOI:** 10.1093/gigascience/gix038

**Published:** 2017-05-20

**Authors:** Xiangqun Zheng-Bradley, Ian Streeter, Susan Fairley, David Richardson, Laura Clarke, Paul Flicek, the 1000 Genomes Project Consortium

**Affiliations:** European Molecular Biology Laboratory, European Bioinformatics Institute, Wellcome Genome Campus, Hinxton, Cambridge CB10 1SD, UK

**Keywords:** alignment, reference genome, GRCh38, sequence reads, read mapping

## Abstract

The 1000 Genomes Project produced more than 100 trillion basepairs of short read sequence from more than 2600 samples in 26 populations over a period of five years. In its final phase, the project released over 85 million genotyped and phased variants on human reference genome assembly GRCh37. An updated reference assembly, GRCh38, was released in late 2013, but there was insufficient time for the final phase of the project analysis to change to the new assembly. Although it is possible to lift the coordinates of the 1000 Genomes Project variants to the new assembly, this is a potentially error-prone process as coordinate remapping is most appropriate only for non-repetitive regions of the genome and those that did not see significant change between the two assemblies. It will also miss variants in any region that was newly added to GRCh38. Thus, to produce the highest quality variants and genotypes on GRCh38, the best strategy is to realign the reads and recall the variants based on the new alignment. As the first step of variant calling for the 1000 Genomes Project data, we have finished remapping all of the 1000 Genomes sequence reads to GRCh38 with alternative scaffold–aware BWA-MEM. The resulting alignments are available as CRAM, a reference-based sequence compression format. The data have been released on our FTP site and are also available from European Nucleotide Archive to facilitate researchers discovering variants on the primary sequences and alternative contigs of GRCh38.

## Data description

### Background

The 1000 Genomes Project Consortium collected and sequenced more than 2600 samples from 26 populations between 2008 and 2013 in order to produce a deep catalogue of human genomic variation. All collected samples were sequenced with two strategies: low-coverage whole genome sequencing (WGS) and whole exome sequencing (WES). Sequence reads were aligned to the GRCh37 human reference genome assembly, and various algorithms were used to make variant calls from the data. The project released variant calls in phases; the final release included 85 million variants of various types and phased haplotypes for those variants [1]. The data set has been widely used by the science community for genotype imputation and many other applications [2].

The Genome Reference Consortium (GRC) released the updated GRCh38 version of the human reference assembly in late 2013. This was the first major update to the reference genome (i.e., one that changes chromosome coordinates) since 2009 [3]. Major improvements in this new release include:

- Correcting erroneous bases, updating the tiling path in highly variable regions, and closing sequence gaps.
- Introducing centromere sequence to replace mega-base stretches of Ns in earlier assemblies. The centromeres are created from a model of the estimated number and order of centromeric repeats.
- Substantially increasing the number of alternative loci associated with the assembly. Following the assembly model introduced with GRCh37 that also supported updates and patches, GRCh38 introduced 261 alternative scaffolds (ALT) to represent diverse haplotypes in 178 chromosomal regions.

With the release of the new assembly, dbSNP lifted all the 1000 Genomes variants—as well as the rest of the data in the archive—to GRCh38 coordinates, and these are distributed on the 1000 Genomes FTP site [4]. This remapping is based on a whole genome alignment between GRCh37 and GRCh38 and is expected to be highly accurate for sites found in regions of the genome that did not change between assembly versions. However, variant sites in repetitive regions or regions that saw sequence or structural changes may be placed inaccurately or not be placed at all. The remapping approach will also be ineffective for any variants that should have been called in regions of the genome missing from the previous reference, and the absence of these regions may have led to mismapping of reads and false positives [5]. To address these potential problems and to create the best possible representation of the 1000 Genomes data on the GRCh38 assembly, we will recall variants and genotypes based on a new underlying read mapping rather than simply distributing the results of a variant lift over.

The first step in recalling the 1000 Genomes variants is mapping the reads to the new reference genome. For the alignment, we chose a new version of BWA-MEM that is ALT-aware and can take advantage of the complete GRCh38 reference genome, rather than just the primary chromosomes [6]. The alignments were improved using the same steps as the 1000 Genomes Project pipeline (base quality recalibration, indel realignment, and duplicate marking), then converted into CRAM format to reduce the disk footprint of the alignments. CRAM uses a reference-based compression approach, resulting in significantly smaller files [7]; in our data set, the average size for CRAMs is 28% of that of corresponding BAMs. Our parameterization of CRAM is considerably more efficient than the generic compression scheme represented by BAM, which is effectively gziped SAM format. These alignments represent the first large-scale open data set in this format and should be a useful resource for community efforts to adapt tools to the CRAM format.

A major use of this alignment data set is variant discovery across all GRCh38 sequences. Compared to the previous 1000 Genomes alignment releases, a unique feature of this data set is read mapping to ALT contigs and human leukocyte antigen (HLA) sequences, facilitating variant discovery and analysis of the ALT sequences and better HLA typing. There are many other possible uses, such as evaluating genome accessibility. We have deposited the CRAM files into European Nucleotide Archive (ENA) to make this resource widely available.

### Methods

#### Preparation of the input files

The methods used for sample collection, library construction, and sequencing are described in the previous 1000 Genomes Project publications [1, 8, 9]. The sequence reads used for the alignments were retrieved from ENA as FASTQ files; sample metadata such as study names, population, and alignment results are listed in Supplemental Table S1. The ReseqTrack software [10] was used to access metadata from ENA using the ReseqTrack script load_from_ena.pl and was also used for file and metadata tracking throughout the alignment process. The GRCh38 alignments used the same criteria as the final phase of the 1000 Genomes Project to select the read data for analysis, namely only sequence data generated by Illumina sequencing and only reads longer than 70 bp (WGS) and 68 bp (WES). All files were verified to be in valid FASTQ format. A complete list of input sequence data and sequence runs used in the alignment can be found on our FTP site [11].

#### Alignment of reads to reference genome

The full analysis set of GRCh38 (accession GCA_000001405.15) was used for this alignment. This includes the primary GRCh38 sequences (autosomes and chromosome X and Y), mitochondria genome, un-localized scaffolds that belong to a chromosome without a definitive location and order, unplaced scaffolds that are in the assembly without a chromosome assignment, the Epstein-Barr virus (EBV) sequence (AJ507799.2), ALT contigs, and the decoy sequences (GCA_000786075.2). The decoy and EBV sequences are not part of the human genome assembly, but they are included in the analysis set to serve as read mapping “sinks” for highly repetitive sequences that are difficult to align and foreign reads that are often present in sequencing samples. In addition to GCA_000001405.15, more than 500 HLA sequences were included as part of the reference assembly to help HLA typing. The alignment target reference data set was unpacked from bwakit-0.7.12 [12], developed by Heng Li, and is available on the 1000 Genomes FTP site [13].

Aligning to the complete GRCh38 reference assembly must allow multiple mappings to accommodate ALT sequences; otherwise BWA-MEM’s random assignment of reads to 1 possible location would lose information. These ALT contigs are given chromosomal context through alignment to the primary reference. The ALT contigs represent 109 Mb of sequence, much of which is near identical to the primary reference. The initial mapping gives these multi-mapping reads a mapping quality of 0. The ALT-aware version of BWA-MEM adjusts the mapping quality for such reads across the non-redundant primary sequence as a post-processing step. It also records the alignments as separate lines in the output BAM files rather than in the XA tag of a primary alignment location. Thus variants on ALT contigs can be used in variant calling independently from the primary sequences.

**Figure 1:**
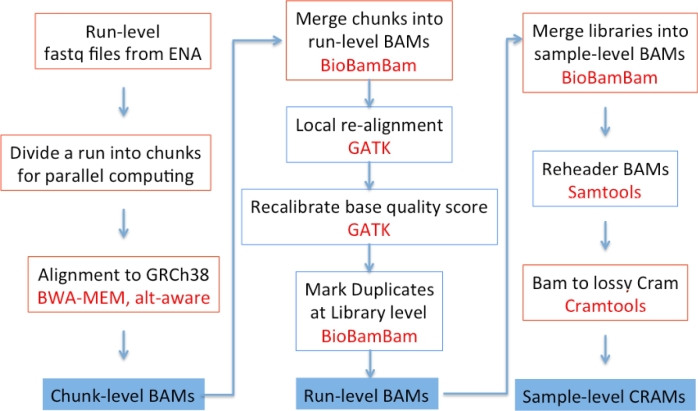
The alignment pipeline flow chart.

Our alignment pipeline was run in a high-throughput compute environment managed using the eHive workflow system [14]. The pipeline split sequence run-level FASTQ files into chunks with a maximum size of 5 million reads to ensure high efficiency. Sequence reads were aligned to GRCh38 chunk by chunk (Fig. 1, left panel) using the following command:

‘bwa mem -t 1 -B 4 -O 6 -E 1 -M -R $rg_string $reference_fasta_file $fastq_file(1) $fastq_file(2) | k8 bwa-postalt.js -p $prefix_hla_hit $reference_fasta_file.alt | samtools view -1 - > $bam_file‘

Subsequently, chunk-level BAMs were sorted and merged into run-level BAMs using BioBamBam [15]. Sequence reads from low coverage WGS and WES were aligned to GRCh38 separately.

#### BAM improvements

BAM improvement steps were run to ensure the alignments were suitable for variant calling purposes (Fig. 1, middle panel). The 1000 Genomes Project included sequencing data from different sequence centers and different versions of the Illumina platform. To manage this heterogeneity, the 1000 Genomes Project developed a base quality recalibration method to reduce center/sequencing machine-specific bias [16], and this was applied to both phase 1 and phase 3 of the 1000 Genomes Project alignments. To recalibrate the aligned base qualities, we used GATK with dbSNP release 142 as the known SNPs. Command lines are as follows:

‘java $jvm_args -jar GenomeAnalysisTK.jar -T BaseRecalibrator -nt 1 -l INFO -cov ReadGroupCovariate -cov QualityScoreCovariate -cov CycleCovariate -cov ContextCovariate -R $reference_fasta -o $recal_data.table -I $bam_file -knownSites $known_snps_from_dbSNP142‘
‘java $jvm_args -jar GenomeAnalysisTK.jar -T PrintReads -l INFO -R $reference_fasta -o $recalibrated_bam -I $bam_file -BQSR $recal_data.table –disable_bam_indexing‘

The 1000 Genomes Project also discovered an excess of false positive variant calls near indels due to alignment parameters that favor mismatches over gaps. The GATK package “IndelRealigner” was developed to address this issue and improve alignments around indels. We used two sets of known indels, mapped to GRCh38 coordinates, for this process: (i) the 1000 Genomes Project phase 3 indels produced by Shapeit2 with coordinates lifted to GRCh38 by NCBI's Remapper [17]; and (ii) the Mills and Devine's indel set [18], lifted to GRCh38 by CrossMap [19] and provided by Alison Meynert from IGMM in Edinburgh (personal communication). Indel realignment used the following command:

*‘java $jvm_args -jar GenomeAnalysisTK.jar -T IndelRealigner -R $reference_fasta -I $bam_file -o $*realigned*_bam_file -targetIntervals $intervals_file -known $known_indels_file(s) -LOD 0.4 -model KNOWNS_ONLY -compress 0 –disable_bam_indexing‘*

Lastly, polymerase chain reaction (PCR)–introduced duplicates were marked at library level using the “markduplicates” function in BioBamBam using the following command line:

‘bammarkduplicates I = $input_bam O = $output_bam index = 1 rmdup = 0‘

After improvement, the run-level BAMs were sorted and merged into sample-level BAMs.

#### Compressing BAMs to CRAMs

The improved, sample-level BAMs were then compressed for distribution to lossy CRAMs using cramtools 3.0.

CRAM is a reference-based compression scheme designed for DNA sequence data and initially described by His-Yang Fritz et al. in 2011 [7]. Briefly, sequences are aligned to a well-established reference assembly, and, rather than storing every aligned base, only bases that are different from the reference are stored. Further file size reduction is achieved by specific lossy techniques in which quality scores, read names, and other alignment tags are stored at a lower resolution or dropped. CRAM is natively supported by HTSlib [20] and Picard [21], as well as the Java toolkit CRAMTools. The format is also accepted by the ENA as a sequencing data format and is being routinely submitted to the archive.

In the CRAM files for the 1000 Genomes GRCh38 alignments, the quality score resolution was reduced by modifying the initial score distribution to one based on the current Illumina 8-bin scheme [22]. Indeed, all data reduction in the creation of the CRAM files was done in a controlled manner using the command line below to ensure no negative impact on downstream variant calling. Given the support of the major NGS toolkits and sequence archives described above, these data present an ideal opportunity for the community to move to this newer, more space efficient, format.

‘java cramtools-3.0.jar cram –input-bam-file $input_bam –output-cram-file $output_cram –capture-all-tags –ignore-tags OQ:CQ:BQ –preserve-read-names –lossy- quality-score-spec ^*^8 –reference-fasta-file $reference_fasta‘

**Table:** 

Software	Installation instructions	Codebase
eHive	http://www.ensembl.org/info/docs/api/api_git.html	https://github.com/Ensembl/ensembl-compara
ReseqTrack	https://github.com/EMBL-EBI-GCA/reseqtrack/blob/master/docs/ alignment_pipeline.txt	https://github.com/EMBL-EBI-GCA/reseqtrack
BWA-MEM	https://github.com/lh3/bwa/blob/master/bwakit/README.md	https://github.com/lh3/bwa
BioBamBam	https://github.com/gt1/biobambam2/blob/master/README.md	https://github.com/gt1/biobambam2
GATK	https://www.broadinstitute.org/gatk/download/	https://github.com/broadgsa/gatk-protected/
CRAMTools	http://www.ebi.ac.uk/ena/software/cram-toolkit	https://github.com/enasequence/cramtools

#### Code availability

The eHive pipeline management software, the ReseqTrack file and metadata tracking software, and the pipeline components for every part of the multi-step alignment process (see Fig. 1) are available for download. Running in parallel on a high-throughput compute cluster is required to ensure completion in a reasonable timeframe.

### Technical validation

To ensure the alignments are high quality, we characterized them and made comparisons between these alignments and the final alignments produced by the 1000 Genomes Project on GRCh37.

#### Comparison to 1000 Genomes phase 3 alignments

The final phase 3 alignments used a very similar pipeline, including the use of a mapping reference comprising human decoy sequence to reduce the rate of mismapped reads and miscalled variants. The phase 3 alignment to GRCh37 was performed using standard BWA v. 0.5.9, and, similar to the process described above, the alignments underwent base quality recalibration, indel realignment, and duplicate removal. Thus, the first phase 3 alignments are a tested, high-quality data set [1].

As summarized in Table 1, the alignment pipeline started with 63 744 gigabases of low-coverage sequence and 28 152 gigabases of WES sequence. The total amounts of aligned sequence in the final CRAM files are slightly larger—66 437 and 30 901 gigabases, respectively—because some of the reads are mapped to multiple locations such as the primary chromosomal region and its corresponding ALT contig. A higher percentage of reads mapped to GRCh38 (96.2% for low-coverage WGS and 97.5% for WES) compared to GRCh37 (92.6% and 93.6%). The percentage of duplicated bases in the GRCh38 alignments are also lower than those of GRCh37 alignment: 3.6% versus 4.1% for the low-coverage and 11.9% versus 13.1% for WES. This difference is likely due to a combination of the improved assembly and a different software package for marking PCR duplications (BioBamBam here and GATK for GRCh37). The coverage statistics noted above and presented in Table 1 were calculated using the GATK calculateHsMetrics function and are very similar for both GRCh37 and GRCh38.

**Table 1:** Characteristics of the GRCh38 alignments

	Low coverage WGS	WES
Sample count	2691 (2535)	2692 (2535)
Total bases (Gbp)	63 744 (60 530)	28 152 (26 571)
Total aligned bases (Gbp)	66 437 (63 783)	30 901 (28 297)
Percentage mapped	96.2 (92.6)	97.5 (93.6)
Percentage PCR duplicated	3.6 (4.1)	11.9 (13.1)
Mapped coverage	8.2 (7.8)	3.8 (3.5)
Mean target coverage	N/A	101.09 (104.72)
%target base 20X	N/A	84.4 (87.24)
CRAM file size (terabytes)	21.2	9.3

Some metrics are presented in comparison with the 1000 Genomes Project phase 3 alignments to the GRCh37 assembly (numbers in parentheses). Mapped coverage was calculated using a nominal 3 Gb genome size.

Taken together, these results suggest the GRCh38 alignment data described here are largely comparable to the tested, high-quality alignment to GRCh37.

#### Mapping quality and read depth

**Figure 2:**
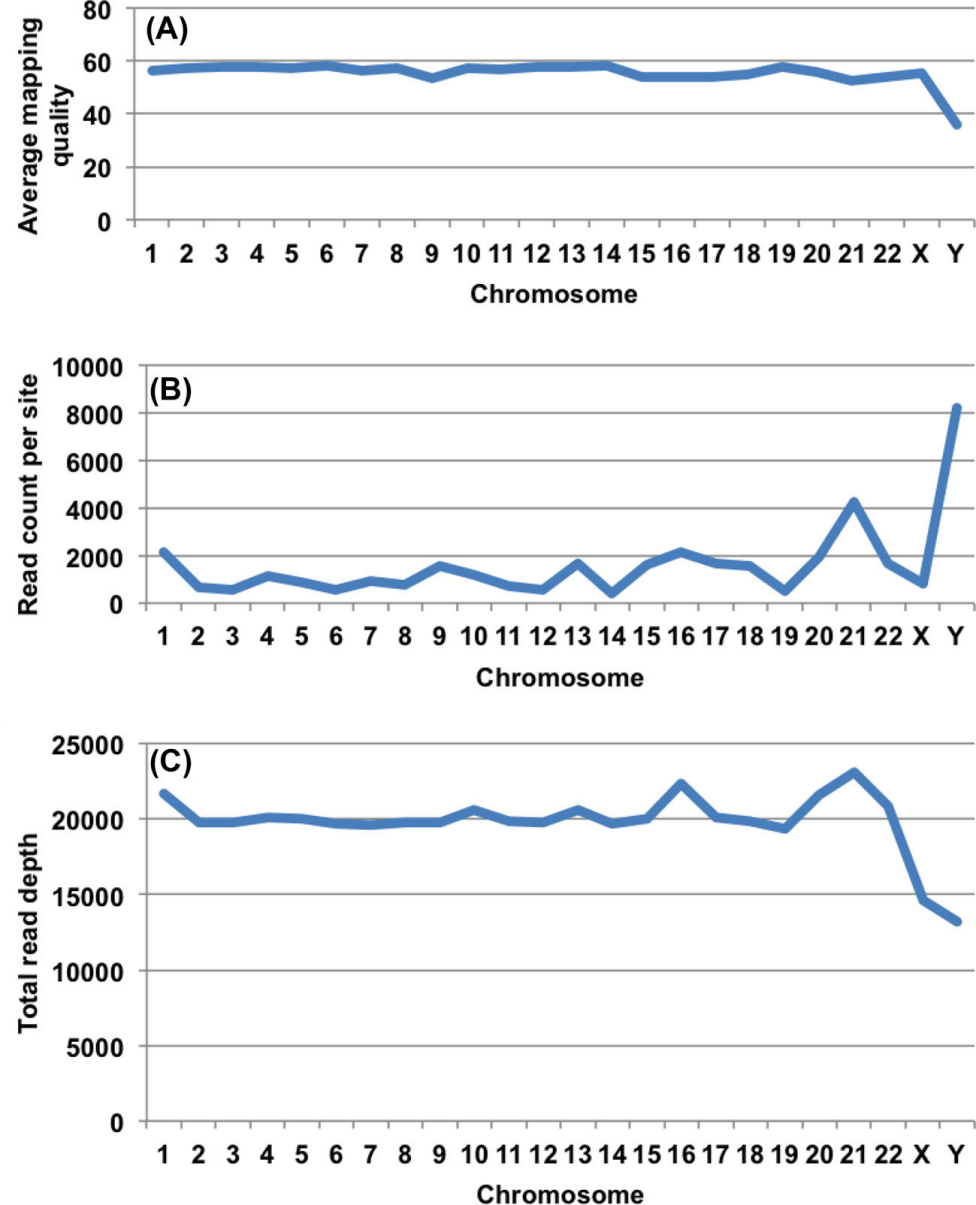
Measurements of mapping quality and total read depth by chromosome for the low-coverage WGS sequence. (**A)** Average mapping quality across all samples. (**B)** Total read count per site with mapping quality of 0 across all samples. (**C)** Total read depth in all samples.

We analyzed mapping quality and total read depth for the low-coverage WGS across chromosomes using bamUtil (Fig. 2) [23]. Except for chromosome Y, the mapping quality is very similar across all chromosomes (Fig. 2A). The lower value on chromosome Y is mainly due to larger than average number of hits with mapping quality 0 (Fig. 2B). This, in turn, is due to the chromosome Y sequence, which contains long stretches of palindromic repetitive sequences [24], and reads mapping to multiple locations are assigned mapping quality of 0.

**Figure 3:**
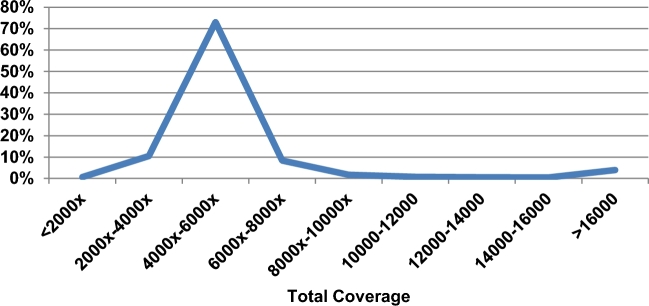
Percentage of sites on chromosome Y by total coverage, showing the expected peak at approximately ×5000.

The total read depth of all samples is plotted by chromosome in Fig. 2C. For autosomes, the mapped reads from more than 2600 samples result in an average total depth of 20 360×, with very small variations (Fig. 2C). The sex ratio in the sample collection is 51:49 female to male, which should result in a total depth for the non–pseudo autosomal regions (non-PAR) of the sex chromosomes of approximately three-quarters of the autosome depth on the X chromosome and one-quarter of the autosome depth on chromosome Y. However, the observed read depths are 14 622× and 13 180× for X and Y, respectively, which is close to the expected 15 000× for the non-PAR region of chromosome X, but much higher than the 5000× expected for chromosome Y (Fig. 2C). An analysis across the length of the Y chromosome shows that the majority (70%) of Y is between 4000×–6000×, with only 6% covered at 10 000× or higher (Fig. 3), meaning that reporting median coverage on Y, although non-standard, would have given more reasonable results. This skewed average coverage is also linked to the repetitive sequences found in chromosome Y. Chromosome 21 also has an enrichment of sites with a mapping quality of 0 (Fig. 2B) and slightly higher read depth compared to the other autosomes (Fig. 2C).

#### Creation and analysis of accessibility masks

We used the results of the bamUtil analysis above to determine which regions of the GRCh38 assembly are accessible for accurate variant calling by short read sequencing. Accessible regions have a combination of reasonable total read depth and mapped reads with reasonable mapping quality. The mean depth across all samples (20 360×) and the percentage of mapping quality 0 reads was used to determine what is considered “accessible.”

Two different genome accessibility masks were produced in the same manner as the final GRCh37 alignments and using the same criteria as those masks. The pilot mask followed the same standards as the 1000 Genomes pilot analysis [9], allowing between a 2-fold change in coverage (i.e., coverage between 10 180× and 40 720×) required 20% or fewer reads with a mapping quality of 0. The strict mask, which was used for the 1000 Genomes phase 3 analysis [1], accepted coverage values between 10 180× and 30 540× and fewer than 0.1% of reads with a mapping quality of 0. The strict mask carried the additional criteria that all accessible base positions have average or higher mapping quality, in this instance 56, a value based on the autosomes.

Comparing the accessibility results (Table 2), GRCh38 has more accessible bases for both masks than GRCh37: 89.0% vs 88.2% using the pilot mask and 74.1% versus 70.5% using the strict mask. Additionally, GRCh38 has fewer bases in the assembly marked N, 5.3% versus 7.7%, as a result of the 60 Mb of GRCh37 gaps that were filled or closed in the new assembly.

**Table 2:** Comparison of GRCh37 and GRCh38 genome accessibility masks

	N	L	H	Z	Q	P
GRCh37-strict	7.66%	1.13%	0.55%	17.20%	2.98%	70.49%
GRCh37-pilot	7.66%	1.13%	0.24%	2.74%		88.23%
GRCh38-strict	5.33%	1.44%	1.04%	18.07%	0.03%	74.09%
GRCh38-pilot	5.33%	1.44%	0.56%	3.67%		89.00%

H: accumulative read depth too high; L: accumulative read depth too low; N: bases that are “N”; P: sites passed the accessibility test; Q: mapping quality less than cutoff; Z: too many reads with mapping quality 0.

We categorized sites in the genome that were masked by whether the base's coverage was too low (L), too high (H), had too many mapping quality 0 reads (Z) or—for the strict mask only—didn’t meet the average mapping quality criteria (Q). For the majority of these categories, the GRCh38 alignment is comparable with the GRCh37 alignment. In both cases, the major reason for a base to be in the strict mask but not the pilot mask was reads with a mapping quality of 0 (Table 2). The largest difference between the alignments is the percentage of sites that failed because the mapped reads have a mapping quality smaller than the cutoff in the strict mask, which dropped from 3% in the GRCh37 mask to 0.03% in the GRCh38 mask. This may be due to the post-processing steps taken by BWA-MEM to adjust mapping quality for reads mapping to both the primary reference and the alternative sequence. Regardless, the accessibility mask creation and analysis suggests that the GRCh38 alignments are as good as, if not better than, the GRCh37 alignments when comparing on the basis of alignment depth.

The masked genomic sequences used in this comparison can be found on the 1000 Genomes FTP site (GRCh38 mask) [25]; (GRCh37 mask) [26].

### Usage notes

CRAM is a relatively new standard data format, and we have included some tips about using these files effectively.

#### Create a local cache of the reference genome, in this case GRCh38, to increase performance

CRAM saves space compared to BAM, in part by removing any reference base from the SAM records. Thus, HTSlib and other tools must have access to the reference sequence when necessary to present alignment records. A local cache of the reference sequence will significantly speed up this process. Indeed, HTSlib and other tools look first to a local cache, then to the central CRAM reference registry, to try and find the correct piece of sequence. This is done using MD5 or SHA1 checksums and, in the case of the reference registry, using the following URL structure:

www.ebi.ac.uk/ena/cram/md5/<hashvalue>
www.ebi.ac.uk/ena/cram/sha1/<hashvalue>

SAMtools can create a local copy of this cache and remove the need to download the data the first time a read sequence is read by any of the tools. We summarize the process below, and more information about it is available [27].

- Download GRCh38 reference FASTA file from the 1000 Genomes FTP site [28].
- Run seq_cache_populate.pl (provided in the standard SAMtools installation) to convert the reference FASTA into a directory tree with the reference sequence MD5 checksums.
  ‘perl samtools/misc/seq_cache_populate.pl-root/path/to/cache/path/to/GRCh38_full_analysis_set_plus_decoy_hla.fa’
(C) Set the following environment variables needed by HTSlib and CRAMTools in order to read the cached genome. The CRAM reference registry is then only used if the given checksum is not found in the cache location.
  ‘export REF_PATH =/path/to/cache/%2s/%2s/%s: http://www.ebi.ac.uk/ena/cram/md5/%s’
  ‘export REF_CACHE =/path/to/cache/%2s/%2s/%s’

By default, SAMtools and CRAMtools first check the reference MD5 sums (@SQ “M5” auxiliary tag) in the directory pointed to by $REF_PATH environment variable. If this is not available, they fall back to querying the CRAM reference genome server at EMBL-EBI and, if neither these are found, to the @SQ “UR” field that contains the URI of sequences.

Once these steps above are finished, the local cache is ready to be used to query data from a CRAM file.

#### Extracting data from CRAM files

CRAM files can be read and processed via Java and C APIs and various supporting tools. Example commands to view CRAM files or convert them to BAM are provided below.

Example: view chr22:1000000-1500000 from CRAM file using

‘*samtools view $input.cram -h chr22*:1000000-1500000 | *less*’

Example: convert CRAM file to BAM file using CRAMtools:

‘java -jar cramtools-3.0.jar bam -I $input.cram -R $reference.fa -O $output.bam’

### Additional file

alignment_1kg_reads_to_GRCh38.supplemental table 1.

### Abbreviations

ALT: alternative scaffold; EBV: Epstein-Barr virus; ENA: European Nucleotide Archive; GRC: Genome Reference Consortium; HLA: human leukocyte antigens; PAR: pseudo autosomal region; PCR = polymerase chain reaction; WES: whole exome sequence; WGS: whole genome sequence.

### Funding

This work was funded by Wellcome Trust (grant numbers WT085532, WT095908, and WT104947) and the European Molecular Biology Laboratory.

### Availability of supporting data

All CRAM files supporting the results of this article are available in ENA and assigned accessions at both study and file levels. Study ERP013771 is the low-coverage WGS data set, which contains 2691 analyses with accessions in the format of ERZnnnnnn, each analysis corresponding to 1 sample-level CRAM file. Similarly, study ERP013770 is the WES data set of 2692 samples, 1 sample-level CRAM file for 1 analysis. All information is summarized in Supplemental Table S1.

### Competing interests

P.F. is a member of the Scientific Advisory Board for Fabric Genomics, Inc.

### Ethics approval and consent to participate

All genome sequence data from the 1000 Genomes Project is consented for open analysis, publication, and distribution. Samples, consent, and ethics details are described in the previous 1000 Genomes Project publications [1, 7, 8].

### Author contributions

X.Z.-B. carried out the remapping and most of the downstream analysis; I.S. developed the original FASTQ retrieval and eHive alignment pipelines, which were adapted by X.Z.-B. to work with the 1000 Genomes Project specifications and output CRAM files; S.F. compared the statistics of the alignment data on GRCh37 and GRCh38; D.R. developed modules to generate XML for data submission to ENA; L.C. and P.F. provided project management, guidance, and ideas; X.Z.-B., L.C., and P.F. wrote the paper.

## Supplementary Material

GIGA-D-17-00028_Original-Submission.pdf

GIGA-D-17-00028_Revision-1.pdf

Response-to-Reviewer-Comments_Original-Submission.pdf

Reviewer-1-Report-(Original-Submission).pdf

Reviewer-2-Report-(Original-Submission).pdf

Reviewer-3-Report-(Original-Submission).pdf

Reviewer-4-Report-(Original-Submission).pdf

Table S1
