# Supplementary material for: Alignment of 1000 Genomes Project reads to reference assembly GRCh38
Source: Gigascience. 2017 May 20;6(7):1–8. doi: 10.1093/gigascience/gix038 (PMC5522380; doi:10.1093/gigascience/gix038)
Supplement: GIGA-D-17-00028_Revision-1.pdf [file gix038_giga-d-17-00028_revision-1.pdf]

GigaScience

Alignment of 1000 Genomes Project Reads to Reference Assembly GRCh38

--Manuscript Draft--

**Manuscript Number:**	GIGA-D-17-00028R1
**Full Title:**	Alignment of 1000 Genomes Project Reads to Reference Assembly GRCh38
**Article Type:**	Data Note
**Funding Information:**	Wellcome Trust (WT085532) — Dr. Paul Flicek<br>Wellcome Trust (WT095908) — Dr. Paul Flicek<br>Wellcome Trust (WT104947) — Dr. Paul Flicek
**Abstract:**	Background<br>The 1000 Genomes Project produced more than 100 trillion basepairs of short read sequence from more than 2600 samples in 26 populations over a period of five years. In its final phase, the project released over 85 million genotyped and phased variants on human reference genome assembly GRCh37. An updated reference assembly, GRCh38, was released in late 2013, but there was insufficient time for the final phase of the project analysis to change to the new assembly. Although it is possible to lift the coordinates of the 1000 Genomes project variants to the new assembly, this is a potentially error prone process as coordinate remapping is most appropriate only for non-repetitive regions of the genome and those that did not see significant change between the two assemblies. It will also miss variants in any region that was that was newly added to GRCh38. Thus, to produce the highest quality variants and genotypes on GRCh38, the best strategy is to realign the reads and recall the variants based on the new alignment.<br><br>Findings<br><br>As the first step of variant calling for the 1000 Genomes Project data, we have finished remapping all of the 1000 Genomes sequence reads to GRCh38 with ALT-aware BWA-MEM. The resulting alignments are available as CRAM, a reference-based sequence compression format.<br><br>Conclusions<br><br>The data have been released on our FTP site and are also available from European Nucleotide Archive (ENA) to facilitate researchers discovering variants on the primary sequences and alternative contigs of GRCh38.
**Corresponding Author:**	Paul Flicek<br><br>UNITED KINGDOM
**Corresponding Author Secondary Information:**	
**Corresponding Author's Institution:**	
**Corresponding Author's Secondary Institution:**	
**First Author:**	Xiangqun Zheng-Bradley
**First Author Secondary Information:**	
**Order of Authors:**	Xiangqun Zheng-Bradley<br>Ian Streeter<br>Susan Fairley

Powered by Editorial Manager® and ProduXion Manager® from Aries Systems Corporation

	David Richardson
	Laura Clarke
	Paul Flicek
	1000 Genomes Project Consortium
**Order of Authors Secondary Information:**	
**Response to Reviewers:**	We thank the reviewers for their careful reading of the manuscript and helpful comments.<br><br>Reviewer 1:<br>The authors described in details how they re-aligned 1000 genome reads to the most recent human reference genome¬-GRCh38. This is not a trivial job given the large data volume. Although the workflow is similar to that used to map reads to GRCh37, it took immense computing resources to generate the resulting CRAM alignment files, and these CRAM files have great reuse potential.<br>Some minor concerns:<br>1) In Table 1, the authors should include "Total aligned bases (Gbp)" from GRCh37 for comparison.<br><br>Added; together with number of samples for GRCh37 and total bases.<br><br>2) In page 9, line53, the authors used PAR abbreviation without explanation.<br><br>Added in line and in List of Abbreviations.<br><br>3) Why Mitochondria chromosome was not included in Figure 2?<br><br>This is a technical issue caused by the software used. Figure 2 was based on numbers calculated by BamUtils, which does not produce numbers for MT.<br><br>4) In each panel of Figure 2, it would be helpful to include another line calculated from GRCh37 for side-by-side comparison.<br><br>We agree that this would be a useful comparison, but the computational requirements of calculating the numbers for the comparison are surprisingly large. Specifically, for GRCh38 BAMs, it took more than 3 weeks of time on our compute farms to get the stats for Fig 2. Each BAM was processed by BamUtils a quality matrix and then the individual matrices were merged into a matrix containing information for all BAMs. The merge step was challenging because the matrix was too big to merge all 2600 BAMs at once and so were repeatedly merged 10 matrices at a time until all merged into one.<br><br>Running the calculation for the GRCh37 BAMs would require all of the steps above plus an additional step of copying all of the BAMs into the scratch space accessible by our high memory machines. Given, this we feel that the value added to the manuscript would not be worth the effort.<br><br>5) In Figure 2C, using the median read depth (on Y-axis) across samples would be more informative and less biased by the respective sequences of the Y-chromosome.<br><br>It is true that using a median read depth in Figure 2C may bring the depth on chrY closer to expected values. However, total read depth is a standard hallmark for evaluation the outcome of any sequencing projects. It will be hard to compare to other studies if we use median coverage here. We have added a statement to the manuscript to make this point.<br><br>6) It would be helpful to other users (and the authors themselves when newer reference genome becomes available) if they could automate the whole process described in Figure 1 and make the code public available.<br><br>We have automated the process internally for use with new samples or updates to the reference genome. The goal of our analysis (and this data note) is to produce and

Powered by Editorial Manager® and ProduXion Manager® from Aries Systems Corporation

	release alignments rather than to produce and release an analysis pipeline. We would be happy to release the code using a permissive open source license (Apache v 2.0) in the interests of full transparency, but it would not run in any other system without major additional development and we do not have the capacity to either do this development ourselves or provide support for external groups starting from our code to modify it for use in another environment.<br><br>Reviewer 2:<br>This is a clear, well-written manuscript describing the realignment of read data from the 1000 Genomes Project to an analysis set including GRCh38, an updated version of the human reference genome assembly. This mapping task is a necessary step in the larger task of re-calling variants on the newer assembly, which the authors cite as their intention. The realignment detailed here will provide for more robust variant calling on GRCh38 and better data interpretation than variant remapping, which is compromised in regions of the assembly that have undergone change and no longer align well between versions. I support the publication of this manuscript, provided the following minor criticisms are addressed:<br>1. p. 3, line 35 (Bullet point #) "Substantially increase the number of alternative haplotypes associated with the assembly" The word "haplotypes" should be modified, as the Genome Reference Consortium did not require that the alternate loci scaffolds be haplotype-specific, though it is true that they increase sequence diversity in the reference (see Box 1, https://www.ncbi.nlm.nih.gov/pubmed/21750661).<br>Changed "hyplotypes" to "loci".<br>2. p. 4, line 57 "the Epstein-Barr virus (EBV)sequence, ALT contigs, and the decoy sequences." Provide the INSDC accessions for EBV (AJ507799.2) and the decoy (GCA_000786075.2).<br>Added.<br>3. p. 4, line 56-58 "...but they are included in the reference" The word "reference" is unclear, as it may be interpreted to mean "reference assembly", rather than the alignment target reference. Suggestion: replace "reference" with "analysis set" or similar phrase.<br>Changed "reference" to "analysis set".<br>4. p. 5, line 6 "The reference data set..." Similarly, a suggestion to replace "reference" with "analysis".<br>Changed "The reference data set" to "The alignment target reference data set".<br>5. p. 6, line Provide a URL for the NCBI Remapper. Suggested: https://www.ncbi.nlm.nih.gov/genome/tools/remap.<br>Added<br><br>Reviewer 3:<br>The manuscript describes the data not for remapping of 1000 Genome Project reads to GRCh38. Remapping to the newest reference genome is very useful for users who wish to use the resource for their own research project. Thus, the resource described in the manuscript is very precious for research community. Reviewer checked the URLs described in the manuscript and confirmed that all the URLs are alive now. Improvements (GRCh37 -> GRCh38) of mapping are successfully summarized in Table 1 and readers can easily grasp the difference.<br>Reviewer cannot find any information about the advantage of CRAM. Although reviewer thinks that the compression by CRAM is superior to other generic compression methods (gzip and bzip2), some real data in data size about the comparison of compression by CRAM to that by gzip and/or bzip2 might be of help to readers who wish to switch to use CRAM for the compression of their own data. It would be much more helpful if the CPU time for compression by CRAM is compared to those by gzip and/or bzip.<br>We believe that a comparison of compression performance of different compression methods is out of scope for this manuscript. However, we would point out that BAM is effectively gzip SAM data and, thus, the reduction from BAM to CRAM is a useful proxy for this comparison. We have added this point to the manuscript.<br><br>Minor point: Term 'HLA' is not explained in the main text while it is in the 'List of abbreviations'.<br>Added<br><br>Reviewer 4:

	In "Alignment of 1000 Genomes Project Reads to Reference Assembly GRCh38", Zheng-Bradley et al. describe the process and results of aligning almost 92 terabases of sequencing reads from the 1000 Genomes Project to the latest human genome reference assembly, GRCh38, including 261 alternate haplotype sequences. The magnitude of the data makes it impractical for most groups to perform these alignments on their own systems, so it is a great service to the genomics community that the authors have produced these alignments and provided them freely via the European Nucleotide Archive (ENA). The alignments will make it possible to perform variant calling on GRCh38, which will provide a more complete and robust set of variants on GRCh38 than the current set of variants remapped from GRCh37. The authors have also generated accessibility masks on GRCh38, i.e. estimates of which parts of GRCh38 can be confidently targeted by short read alignments, using the same criteria as were used on GRCh37 by the 1000 Genomes Project. I was able to find CRAM files from the project in ENA easily, and since they are provided along with .crai index files, the CRAM file URLs can be remotely accessed efficiently, e.g. as genome browser custom tracks. The manuscript is well-written. The input data and alignment process are described in detail down to the level of command lines for crucial steps. The tools used are open-source tools and the manuscript includes URLs for installing the tools. The authors provide a good comparison of the new GRCh38 alignments to the pre-existing GRCh37 alignments. I have only minor comments/questions that I would like the authors to address before publication:<br>Can you provide a URL for downloading the accessibility masks?<br>URLs are provided at the top of page 10<br>Methods section 4: HTSlib and Picard are mentioned without references; there should be at least a URL for each.<br>These have been added.<br>Methods section 5: It would be nice to add a brief description of the cluster used (cores, RAM etc) and the number of CPU hours. While those statistics have no bearing on the quality of the alignments, they do give a sense of the resources required for such a large endeavor.<br>In fact, we used different clusters over the course of the analysis, which took several month of wall time and we did not collect metrics of CPU hours during the analysis. Moreover, we do not think the sizes of the clusters provide particularly very useful information because, even though the cluster may have thousands of nodes, each user is allowed to run only a limited number of jobs because of our fair share policy.<br>Technical validation section 1: regarding "The percentage of duplicated bases in the GRCh38 alignments" -- what are duplicated bases? PCR duplicates? Stretches of identical reference bases??<br>PCR duplication. It was mentioned explicitly in the sentence following the question (“marking PCR duplications” was done by BioBamBam).<br>Table 1: what is "Mapped coverage"?<br>Figure 3: It might be helpful to show a longer tail on the right... perhaps that might help to explain how the average coverage of Y is 13180x when it seems from Figure 3 that 90% of sites are between 2000x and 8000x. There must be some sites with extremely high coverage to pull the median of ~5000x up to an average of ~13000x.<br>Extended figure 3 to the right as suggested.<br>Usage notes (or elsewhere): how much space was saved by compressing BAM to CRAM? If it's too late to calculate because the GRCh38 BAM files are gone, at least compare to GRCh37 BAM total size. Since this is the first open CRAM dataset on thousands of genomes, it would be interesting to know how much space was saved.<br>Added “in our dataset, the average size for CRAMs are 28% of that of corresponding BAMs. “ to page 4.<br>typo: "checksum is not founded in": "founded" --> "found"<br>Corrected<br>... "@SQ 'UR' field" -- briefly define "UR"<br><br>Added “which contains URI of sequences” to page 11<br>Availability of supporting data: "one for one analysis" -- is that "one file for one analysis"?<br>Changed to “one sample-level CRAM file for one analysis “<br>Reference typo: The authors of [10] (biobambam) are listed as "Tischler German L, Steven." but I believe it should be "Tischler G, Leonard S".<br>Corrected<br>The first time that the terms "run" and/or "run-level" are used ("input sequence data

Powered by Editorial Manager® and ProduXion Manager® from Aries Systems Corporation

	and runs used in the alignment", "split run-level FASTQ files into chunks"), it might help to say "sequencing run" for context, for example "input sequence data and sequencing runs" and/or "split sequencing run-level FASTQ files into chunks".<br>Corrected as suggested.<br>Thanks for doing the heavy lifting -- these are definitely an improvement over GRCh37 alignments. I am very much looking forward to the freshly called variants! :)
**Additional Information:**	
**Question**	**Response**
Are you submitting this manuscript to a special series or article collection?	No
**Experimental design and statistics**<br><br>Full details of the experimental design and statistical methods used should be given in the Methods section, as detailed in our Minimum Standards Reporting Checklist. Information essential to interpreting the data presented should be made available in the figure legends.<br><br>Have you included all the information requested in your manuscript?	Yes
**Resources**<br><br>A description of all resources used, including antibodies, cell lines, animals and software tools, with enough information to allow them to be uniquely identified, should be included in the Methods section. Authors are strongly encouraged to cite Research Resource Identifiers (RRIDs) for antibodies, model organisms and tools, where possible.<br><br>Have you included the information requested as detailed in our Minimum Standards Reporting Checklist?	Yes
**Availability of data and materials**<br><br>All datasets and code on which the conclusions of the paper rely must be either included in your submission or deposited in publicly available repositories (where available and ethically appropriate), referencing such data using a unique identifier in the references and in the "Availability of Data and Materials" section of your manuscript.<br><br>Have you have met the above	Yes

requirement as detailed in our Minimum Standards Reporting Checklist?	

Manuscript

Click here to download Manuscript Alignment_of_1kg_reads_to_GRCh38.gigaScience.20170501a.d

Alignment of 1000 Genomes Project Reads to Reference Assembly GRCh38

Xiangqun Zheng-Bradley, Ian Streeter, Susan Fairley, David Richardson, Laura Clarke, Paul Flicek and the 1000 Genomes Project Consortium

European Molecular Biology Laboratory, European Bioinformatics Institute, Wellcome Genome Campus, Hinxton, Cambridge CB10 1SD, United Kingdom

Emails:

Zheng_holly@hotmail.com (XZ)
streeter@ebi.ac.uk (IS)
fairley@ebi.ac.uk (SF)
davidr@ebi.ac.uk (DR)
laura@ebi.ac.uk (LC)
flicek@ebi.ac.uk (PF)

Corresponding author: Paul Flicek

Abstract

Background

The 1000 Genomes Project produced more than 100 trillion basepairs of short read sequence from more than 2600 samples in 26 populations over a period of five years. In its final phase, the project released over 85 million genotyped and phased variants on human reference genome assembly GRCh37. An updated reference assembly, GRCh38, was released in late 2013, but there was insufficient time for the final phase of the project analysis to change to the new assembly. Although it is possible to lift the coordinates of the 1000 Genomes project variants to the new assembly, this is a potentially error prone process as coordinate remapping is most appropriate only for non-repetitive regions of the genome and those that did not see significant change between the two assemblies. It will also miss variants in any region that was that was newly added to GRCh38. Thus, to produce the highest quality variants and genotypes on GRCh38, the best strategy is to realign the reads and recall the variants based on the new alignment.

Findings

As the first step of variant calling for the 1000 Genomes Project data, we have finished remapping all of the 1000 Genomes sequence reads to GRCh38 with ALT-aware BWA-MEM. The resulting alignments are available as CRAM, a reference-based sequence compression format.

Conclusions

The data have been released on our FTP site and are also available from European Nucleotide Archive (ENA) to facilitate researchers discovering variants on the primary sequences and alternative contigs of GRCh38.

Keywords

alignment, reference genome, GRCh38, sequence reads, read mapping

Data Description

Background

The 1000 Genomes Project consortium collected and sequenced more than 2600 samples from 26 populations between 2008 and 2013 in order to produce a deep catalogue of human genomic variation. All collected samples were sequenced with two strategies: low coverage whole genome sequencing (WGS) and whole exome sequencing (WES). Sequence reads were aligned to the GRCh37 human reference genome assembly and various algorithms were used to make variant calls from the data. The project released variant calls in phases; the final release included 85 millions variants of various types and phased haplotypes for those variants [1]. The dataset has been widely used by the science community for genotype imputation and many other applications [2].

The Genome Reference Consortium (GRC) released the updated GRCh38 version of the human reference assembly in late 2013. This was the first major update to the reference genome (i.e. one that changes chromosome coordinates) since 2009 [3]. Major improvements in this new release include:

1. Correcting erroneous bases, updating the tiling path in highly variable regions and closing sequence gaps.
2. Introducing centromere sequence to replace mega-base stretches of Ns in earlier assemblies. The centromeres are created from a model of the estimated number and order of centromeric repeats.
3. Substantially increasing the number of alternative loci associated with the assembly. Following the assembly model introduced with GRCh37 that also supported updates and patches, GRCh38 introduced 261 alternative scaffolds (ALT) to represent diverse haplotypes in 178 chromosomal regions.

With the release of the new assembly, dbSNP lifted all the 1000 Genomes variants—as well as the rest of the data in the archive—to GRCh38 coordinates and these are distributed on the 1000 Genomes FTP site (ftp://ftp.1000genomes.ebi.ac.uk/vol1/ftp/release/20130502/supporting/GRCh38_positions). This remapping is based on a whole genome alignment between GRCh37 and GRCh38 and is expected to be highly accurate for sites found in regions of the genome that did not change between assembly versions. However, variant sites in repetitive regions or regions that saw sequence or structural changes may be placed inaccurately or not be placed at all. The remapping approach will also be ineffective for any variants that should have been called in regions of the genome missing from the previous reference and the absence of these regions may have led to mis-mapping of reads and false positives [4]. To address these potential problems and to create the best possible representation of the 1000 Genomes data on the GRCh38 assembly, we will recall variants and genotypes based on a new underlying read mapping rather than simply distribute the results of a variant lift over.

The first step in recalling the 1000 Genomes variants is mapping the reads to the new reference genome. For the alignment, we chose a new version of BWA-MEM that is ALT-aware and can take advantage of the complete GRCh38 reference genome, rather than just the primary chromosomes [5]. The alignments were improved using the same steps as the 1000 Genomes Project pipeline (base quality recalibration, indel realignment and duplicate marking) then converted into CRAM format to reduce the disk footprint of the alignments. CRAM uses a reference-based compression approach resulting in significantly smaller files [6]; in our dataset, the average size for CRAMs are 28% of that of corresponding BAMs. Our parameterization of CRAM is considerably more efficient than the generic compression scheme represented by BAM, which is effectively gziped SAM format. These alignments represent the first large-scale open dataset in this format and should be a useful resource for community efforts to adapt tools to the CRAM format.

A major use of this alignment data set is variant discovery across all GRCh38 sequences. Compared to the previous 1000 Genomes alignment releases, a unique feature of this data set is read mapping to ALT contigs and Human leukocyte antigen (HLA) sequences, facilitating variant discovery and analysis of the ALT sequences and better HLA typing. There are many other possible uses, such as evaluating genome accessibility. We have deposited the CRAM files into ENA to make this resource widely available.

Methods

1. Preparation of the input files

The methods used for sample collection, library construction and sequencing are described in the previous 1000 Genomes Project publications [1, 7, 8]. The sequence reads used for the alignments were retrieved from ENA as FASTQ files; sample meta data such as study names, population, as well as alignment results are listed in Supplemental Table 1. The ReseqTrack software [9] was used to access metadata from ENA using the ReseqTrack script load_from_ena.pl and also used for file and metadata tracking throughout the alignment process. The GRCh38 alignments used the same criteria as the final phase of the 1000 Genomes Project to select the read data for analysis, namely only sequence data generated by Illumina sequencing and only reads longer than 70bps (WGS) and 68bps (WES). All files were verified to be valid FASTQ format. A complete list of input sequence data and sequence runs used in the alignment can be found on our FTP site (ftp://ftp.1000genomes.ebi.ac.uk/vol1/ftp/data_collections/1000_genomes_project/1000genomes.sequence.index)

2. Alignment of reads to reference genome

The full analysis set of GRCh38 (accession GCA_000001405.15) was used for this alignment. This includes the primary GRCh38 sequences (autosomes and

chromosome X and Y), mitochondria genome, un-localized scaffolds which belong to a chromosome without a definitive location and order, unplaced scaffolds which are in the assembly without a chromosome assignment, the Epstein-Barr virus (EBV) sequence (AJ507799.2), ALT contigs, and the decoy sequences (GCA_000786075.2). The decoy and EBV sequences are not part of the human genome assembly but they are included in the analysis set to serve as read mapping "sinks" for highly repetitive sequences that are difficult to align and foreign reads that are often present in sequencing samples. In addition to GCA_000001405.15, more than 500 HLA sequences were included as part of the reference assembly to help HLA typing. The alignment target reference data set was unpacked from bwakit-0.7.12 (https://github.com/lh3/bwa/tree/master/bwakit) developed by Heng Li and is available from ftp://ftp.1000genomes.ebi.ac.uk/vol1/ftp/technical/reference/GRCh38_reference_genome/.

Aligning to the complete GRCh38 reference assembly must allow multiple mappings to accommodate ALT sequences otherwise BWA-MEM's random assignment of reads to one possible location would lose information. These ALT contigs are given chromosomal context through alignment to the primary reference. The ALT contigs represent 109Mb of sequence, much of which is near identical to the primary reference. The initial mapping gives these multi-mapping reads a mapping quality of zero. The alt aware version of BWA-MEM adjusts the mapping quality for such reads across non-redundant primary sequence as a post-processing step. It also records the alignments as separate lines in the output BAM files rather than in the XA tag of a primary alignment location. Thus variants on ALT contigs can be used in variant calling independently from the primary sequences.

Our alignment pipeline was run in a high throughput compute environment managed using the eHive workflow system [10]. The pipeline split sequence run-level FASTQ files into chunks with a maximum size of 5 million reads to ensure high efficiency. Sequence reads were aligned to GRCh38 chunk by chunk (Figure 1 left panel) using the following command:

`bwa mem -t 1 -B 4 -O 6 -E 1 -M -R $rg_string $reference_fasta_file $fastq_file(1) $fastq_file(2) | k8 bwa-postalt.js -p $prefix_hla_hit $reference_fasta_file.alt | samtools view -1 - > $bam_file`

Subsequently, chunk level BAMs were sorted and merged into run-level BAMs using BioBamBam [11]. Sequence reads from low coverage WGS and WES were aligned to GRCh38 separately.

3. BAM improvements

BAM improvement steps were run to ensure the alignments are suitable for variant calling purposes (Figure 1, middle panel). The 1000 Genomes Project included sequencing data from different sequence centers and different versions of the

Illumina platform. To manage this heterogeneity the 1000 Genomes Project developed a base quality recalibration method to reduce center/sequencing machine specific bias [12] and this was applied to both Phase 1 and Phase 3 of the 1000 Genomes Project alignments. To recalibrate the aligned base qualities, we used GATK with dbSNP release 142 as the known SNPs. Command lines are as follows:

`java $jvm_args -jar GenomeAnalysisTK.jar -T BaseRecalibrator -nt 1 -l INFO -cov ReadGroupCovariate -cov QualityScoreCovariate -cov CycleCovariate -cov ContextCovariate -R $reference_fasta -o $recal_data.table -I $bam_file -knownSites $known_snps_from_dbSNP142`

`java $jvm_args -jar GenomeAnalysisTK.jar -T PrintReads -l INFO -R $reference_fasta -o $recalibrated_bam -I $bam_file -BQSR $recal_data.table --disable_bam_indexing`

The 1000 Genomes Project also discovered an excess of false positive variant calls near indels due to alignment parameters that favor mismatches over gaps. The GATK package 'IndelRealigner' was developed to address this issue and improve alignments around indels. We used two sets of known indels, mapped to GRCh38 coordinates, for this process: (i) the 1000 Genomes Project phase3 indels produced by Shapeit2 with coordinates lifted to GRCh38 by NCBI's Remapper (https://www.ncbi.nlm.nih.gov/genome/tools/remap); and (ii) the Mills and Devine's indel set [13], lifted to GRCh38 by CrossMap [14] and provided by Alison Meynert from IGMM in Edinburgh (personal communication). Indel realignment used the following command:

`java $jvm_args -jar GenomeAnalysisTK.jar -T IndelRealigner -R $reference_fasta -I $bam_file -o $realigned_bam_file -targetIntervals $intervals_file -known $known_indels_file(s) -LOD 0.4 -model KNOWNS_ONLY -compress 0 --disable_bam_indexing`

Lastly, PCR-introduced duplicates were marked at library level using "markduplicates" function in BioBamBam using the following command line:

`bammarkduplicates I=$input_bam O=$output_bam index=1 rmdup=0`

After improvement, the run-level BAMs were sorted and merged into sample-level BAMs.

4. Compressing BAMs to CRAMs

The improved, sample-level BAMs were then compressed to lossy CRAMs using cramtools-3.0 for distribution.

CRAM is a reference-based compression scheme designed for DNA sequence data and initially described by His-Yang Fritz *et al.* in 2011 [6]. Briefly, sequences are aligned to a well-established reference assembly and, rather than storing every

aligned base, only bases that are different from the reference are stored. Further file size reduction is achieved by specific lossy techniques in which quality scores, read names and other alignment tags are stored at a lower resolution or dropped. CRAM is natively supported by HTSlib (http://htslib.org) and Picard https://broadinstitute.github.io/picard/), as well as the java toolkit, CRAMTools. The format is also accepted by the ENA as a sequencing data format and is being routinely submitted to the archive.

In the CRAM files for the 1000 Genomes GRCh38 alignments, the quality score resolution was reduced by modifying the initial score distribution to one based on the current Illumina 8-bin scheme (http://www.illumina.com/content/dam/illumina-support/documents/myillumina/e96e90a9-698d-4a0b-9b33-9445c5ad723d/whitepaper_datacompression.pdf). Indeed, all data reduction in the creation of the CRAM files was done in a controlled manner using the command line below to ensure no negative impact on downstream variant calling. Given the support of the major NGS toolkits and sequence archives described above, these data present a ideal opportunity for the community to move to this newer, more space efficient, format.

`java cramtools-3.0.jar cram --input-bam-file $input_bam --output-cram-file $output_cram --capture-all-tags --ignore-tags OQ:CQ:BQ --preserve-read-names --lossy- quality-score-spec *8 --reference-fasta-file $reference_fasta `

5. Code availability

The eHive pipeline management software, the ReseqTrack file and metadata tracking software and the pipeline components for every part of the multi-step alignment process (see Figure 1) are available for download. Running in parallel on a high throughput compute cluster is required to ensure completion in a reasonable timeframe.

Software	Installation Instructions	Codebase
eHive	http://www.ensembl.org/info/docs/api/api_git.html	https://github.com/Ensembl/ensembl-compara
ReseqTrack	https://github.com/EMBL-EBI-GCA/reseqtrack/blob/master/docs/alignment_pipeline.txt	https://github.com/EMBL-EBI-GCA/reseqtrack
BWA-MEM	https://github.com/lh3/bwa/blob/master/bwakit/README.md	https://github.com/lh3/bwa
BioBamBam	https://github.com/gt1/biobambam2/blob/master/README.md	https://github.com/gt1/biobambam2
GATK	https://www.broadinstitute.org/gatk/download/	https://github.com/broadgsa/gatk-protected/
CRAMTools	http://www.ebi.ac.uk/ena/softwar	https://github.com/enaseque

	e/cram-toolkit	nce/cramtools

Technical Validation

To ensure the alignments are high quality, we characterized them and made comparisons between these alignments and the final alignments produced by the 1000 Genomes project on GRCh37.

1. Comparison to 1000 Genomes Phase 3 alignments

The final phase 3 alignments used a very similar pipeline including the use of a mapping reference comprising human decoy sequence to reduce the rate of mis-mapped reads and miscalled variants. The phase3 alignment to GRCh37 was performed using standard BWA v0.5.9 and, similar to the process described above, the alignments underwent base quality recalibration, indel realignment, and duplicate removal. Thus, the first phase 3 alignments are a tested, high-quality dataset [1].

As summarized in Table 1, the alignment pipeline started with 63,744 gigabases of low coverage sequence and 28,152 gigabases of WES sequence. The total amounts of aligned sequence in the final CRAM files are slightly larger—66,437 and 30,901 gigabases, respectively—because some of the reads are mapped to multiple locations such as the primary chromosomal region and its corresponding ALT contig. A higher percentage of reads mapped to GRCh38 (96.2% for low coverage WGS and 97.5% for WES) compared to GRCh37 (92.6% and 93.6%). The percentage of duplicated bases in the GRCh38 alignments are also lower than those of GRCh37 alignment: 3.6% vs. 4.1% for the low coverage and 11.9% vs. 13.1% for WES. This difference is likely due to a combination of the improved assembly and a different software package for marking PCR duplications (BioBamBam here and GATK for GRCh37). The coverage statistics noted above and presented in Table 1 were calculated using GATK calculateHsMetrics function and are very similar for both GRCh37 and GRCh38.

Taken together, these results suggest the GRCh38 alignment data described here is largely comparable to the tested, high quality alignment to GRCh37.

2. Mapping quality and read depth

We analyzed mapping quality and total read depth for the low coverage WGS across chromosomes using BamUtil (Figure 2; http://genome.sph.umich.edu/wiki/BamUtil). Except for chromosome Y, the mapping quality is very similar across all chromosomes (Figure 2a). The lower value on chromosome Y is mainly due to larger than average number of hits with mapping

quality zero (Figure 2b). This, in turn, is due to the chromosome Y sequence, which contains long stretches of palindromic repetitive sequences [15] and reads mapping to multiple locations are assigned mapping quality of zero.

The total read depth of all samples is plotted by chromosome in Figure 2c. For autosomes, the mapped reads from more than 2600 samples result in an average total depth of 20,360x, with very small variations (Figure 2c). The sex ratio in the sample collection is 51/49 female to male, which should result in a total depth for the non-Pseudo Autosomal Regions (non-PAR) of the sex chromosomes of approximately three quarters of the autosome depth on the X chromosome and one quarter of the autosome depth on chromosome Y. However, the observed read depths are 14622x and 13180x for X and Y, respectively, which is close to the expected 15000x for the non-PAR region of chromosome X, but much higher than the 5000x expected for chromosome Y (Figure 2c). An analysis across the length of the Y chromosome, shows that the majority (70%) of Y is between 4000-6000x, with only 6% covered at 10000x or higher (Figure 3) meaning that reporting median coverage on Y, although non-standard, would have given more reasonable results. This skewed average coverage is also linked to the repetitive sequences found in chromosome Y. Chromosome 21 also has an enrichment of sites with mapping quality of zero (Figure 2b) and slightly higher read depth compared to the other autosomes (Figure 2c).

3. Creation and analysis of accessibility masks

We used the results of the BamUtil analysis above to determine which regions of the GRCh38 assembly are accessible for accurate variant calling by short read sequencing. Accessible regions have a combination of reasonable total read depth and mapped reads with reasonable mapping quality. The mean depth across all samples (20,360x) and the percentage of mapping quality zero reads was used to determine what is considered ‘accessible’.

Two different genome accessibility masks were produced in the same manner as the final GRCh37 alignments and using the same criteria as those masks. The pilot mask followed the same standards as the 1000 Genomes pilot analysis [8] allowing between a two fold change in coverage (i.e. coverage between 10,180x and 40,720x) required 20% or fewer reads with mapping quality zero. The strict mask, which was used for the 1000 Genomes phase 3 analysis [1], accepted coverage values between 10,180x and 30,540x and fewer than 0.1% of reads with mapping quality zero. The strict mask carried the additional criteria that all accessible base positions have average or higher mapping quality, in this instance 56; a value based on the autosomes.

Comparing the accessibility results (Table 2), GRCh38 has more accessible bases for both masks than GRCh37: 89.0% vs 88.2% using the pilot mask and 74.1% vs 70.5% using the strict mask. Additionally, GRCh38 has fewer bases in the assembly

marked as N, 5.3% versus 7.7%, as a result of the 60Mb of GRCh37 gaps that were filled or closed in the new assembly.

We categorized sites in the genome that were masked by whether the base's coverage was too low (L), too high (H), had too many mapping quality zero reads (Z) or—for the strict mask only—didn't meet the average mapping quality criteria (Q). For the majority of these categories, the GRCh38 alignment is comparable with the GRCh37 alignment. In both cases, the major reason for a base to be in the strict mask, but not the pilot mask was reads with mapping quality zero (Table 2). The largest difference between the alignments is the percentage of sites failed because the mapped reads have a mapping quality smaller than the cutoff in the strict mask, which dropped from 3% in the GRCh37 mask to 0.03% in the GRCh38 mask. This may be due to the post-processing steps taken by BWA-MEM to adjust mapping quality for reads mapping to both the primary reference and the alternative sequence. Regardless, the accessibility mask creation and analysis suggests that the GRCh38 alignments are as good as, if not better than, the GRCh37 alignments when comparing on the basis of alignment depth.

The masked genomic sequences used in this comparison can be found at
ftp://ftp.1000genomes.ebi.ac.uk/vol1/ftp/data_collections/1000_genomes_project/working/20160622_genome_mask_GRCh38/ (GRCh38 mask)
ftp://ftp.1000genomes.ebi.ac.uk/vol1/ftp/release/20130502/supporting/accessible_genome_masks/ (GRCh37 mask)

Usage Notes

CRAM is a relatively new standard data format and we have included some tips about using these files effectively.

1. Create a local cache of the reference genome, in this case GRCh38, to increase performance

CRAM saves space compared to BAM in part by removing any reference base from the SAM records. Thus, HTSlib and other tools must have access to the reference sequence when necessary to present alignment records. A local cache of the reference sequence will significantly speed up this process. Indeed, HTSlib and other tools look first to a local cache, then the central CRAM reference registry to try and find the correct piece of sequence. This is done using MD5 or SHA1 checksums and in the case of the reference registry, using the following URL structure:

www.ebi.ac.uk/ena/cram/md5/<hashvalue>
www.ebi.ac.uk/ena/cram/sha1/<hashvalue>

SAMtools can create a local copy of this cache and remove the need to download the data the first time a read sequence is read by any of the tools. We summarize the

process below and more information about it is available from http://www.htslib.org/workflow/#mapping_to_cram.

A) Download GRCh38 reference FASTA file from the FTP site

ftp://ftp.1000genomes.ebi.ac.uk/vol1/ftp/technical/reference/GRCh38_reference_genome/GRCh38_full_analysis_set_plus_decoy_hla.fa

B) Run seq_cache_populate.pl (provided in the standard SAMtools installation) to convert the reference FASTA into a directory tree with the reference sequence MD5 checksums.

'perl samtools/misc/seq_cache_populate.pl -root /path/to/cache /path/to/GRCh38_full_analysis_set_plus_decoy_hla.fa'

C) Set the following environment variables needed by HTSlib and CRAMTools in order to read the cached genome. The CRAM reference registry is then only used if the given checksum is not found in the cache location.

'export REF_PATH=/path/to/cache/%2s/%2s/%s:http://www.ebi.ac.uk/ena/cram/md5/%s'

'export REF_CACHE=/path/to/cache/%2s/%2s/%s'

By default SAMtools and CRAMtools first check the reference MD5 sums (@SQ "M5" auxiliary tag) in the directory pointed to by $REF_PATH environment variable. If this is not available, they fall back to querying the CRAM reference genome server at EMBL-EBI and, if neither these are found, to the @SQ "UR" field which contains URI of sequences.

Once these steps above are finished, the local cache is ready to be used to query data from a CRAM file.

2. Extracting data from CRAM files

CRAM files can be read and processed via java and C APIs and various supporting tools. Example commands to view CRAM files or convert them to BAM are provided below.

Example: view chr22:1000000-1500000 from CRAM file using SAMtools (version 1.2 or higher):

*'samtools view $input.cram -h chr22:*1000000-1500000 | *less'*

Example: convert CRAM file to BAM file using CRAMtools:

‘java -jar cramtools-3.0.jar bam -I $input.cram -R $reference.fa -O $output.bam’

Availability of supporting data

All CRAM files supporting the results of this article are available in ENA and assigned accessions at both study and file levels. Study ERP013771 is the low coverage WGS dataset, which contains 2691 analyses with accessions in the format of ERZnnnnnn, each analysis corresponding to one sample-level CRAM file. Similarly, study ERP013770 is the WES dataset of 2692 samples, one sample-level CRAM file for one analysis. All information is summarized in Supplemental Table 1.

List of abbreviations

WGS – whole genome sequence
WES – whole exome sequence
ENA - European Nucleotide Archive
GRC - the Genome Reference Consortium
ALT – alternative scaffold
EBV - the Epstein-Barr virus
PAR – pseudo autosomal regions
HLA – human leukocyte antigens

Ethics approval and consent to participate

All genome sequence data from the 1000 Genomes Project is consented for open analysis, publication and distribution. Samples, consent and ethics details are described in the previous 1000 Genomes Project publications [1, 7, 8].

Consent for publication

Not applicable

Competing interests

P.F. is a member of the Scientific Advisory Board for Fabric Genomics, Inc.

Funding

This work was funded by Wellcome Trust (grant numbers WT085532, WT095908 and WT104947) and the European Molecular Biology Laboratory.

Author contributions

X.Z.-B. carried out the remapping and most of the downstream analysis; I.S. developed the original FASTQ retrieval and eHive alignment pipelines, which were adapted by X.Z.-B. to work with the 1000 Genomes Project specifications and output CRAM files; S.F. compared the statistics of the alignment data on GRCh37 and GRCh38; D.R. developed modules to generate XML for data submission to ENA; L.C. and P.F provided project management, guidance and ideas; X.Z.-B., L.C. and P.F. wrote the paper.

Acknowledgements

The authors acknowledge Shane McCarthy for sharing with us his experience on working with the 1000 Genome Project alignment pipeline; Vadim Zalunin and James Bonfield for their help with CRAM, CRAMTools, and HTSlib; Dylan Spalding for providing coordinate lift data for known indels; Rasko Leinonen for assistance in submitting data the ENA and Heng Li for helpful discussions regarding the ALT-aware BWA-MEM.

References

1. The 1000 Genomes Project Consertium. A global reference for human genetic variation. *Nature* 2015; 526:68-74.
2. Zheng-Bradley X, Flicek P. Applications of the 1000 Genomes Project resources. *Brief Funct Genomics* 2016.
3. Church DM, Schneider VA, Graves T *et al*. Modernizing reference genome assemblies. *PLoS Biol* 2011; 9:e1001091.
4. Schneider VA, Lindsay TG, Howe K *et al*. Evaluation of GRCh38 and de novo haploid genome assemblies demonstrates the enduring quality of the reference assembly. *bioRxiv* 2016.
5. Li H. Aligning sequence reads, clone sequences and assembly contigs with BWA-MEM. 2013; arXiv:1303.3997v2 [q-bio.GN].
6. Hsi-Yang Fritz M, Leinonen R, Cochrane G *et al*. Efficient storage of high throughput DNA sequencing data using reference-based compression. *Genome research* 2011; 21:734-740.
7. The 1000 Genomes Project Consertium. An integrated map of genetic variation from 1,092 human genomes. *Nature* 2012; 491:56-65.
8. The 1000 Genomes Project Consortium. A map of human genome variation from population-scale sequencing. *Nature* 2010; 467:1061-1073.
9. Clarke L, Zheng-Bradley X, Smith R *et al*. The 1000 Genomes Project: data management and community access. *Nat methods* 2012; 9:459-462.
10. Severin J, Beal K, Vilella AJ *et al*. eHive: an artificial intelligence workflow system for genomic analysis. *BMC Bioinformatics* 2010; 11:240.
11. Tischler G, Leonard S. biobambam: tools for read pair collation based algorithms on BAM files. *Source Code Biol Med* 2014; 9:13.
12. DePristo MA, Banks E, Poplin R *et al*. A framework for variation discovery and genotyping using next-generation DNA sequencing data. *Nature genetics* 2011; 43:491-498.
13. Mills RE, Pittard WS, Mullaney JM *et al*. Natural genetic variation caused by small insertions and deletions in the human genome. *Genome research* 2011; 21:830-839.
14. Zhao H, Sun Z, Wang J *et al*. CrossMap: a versatile tool for coordinate conversion between genome assemblies. *Bioinformatics* 2014; 30:1006-1007.
15. Skaletsky H, Kuroda-Kawaguchi T, Minx PJ *et al*. The male-specific region of the human Y chromosome is a mosaic of discrete sequence classes. *Nature* 2003; 423:825-837.

Click here to download Table
Alignment_to_GRCh38.data_note.2017307.tables_figures.docx

Table 1. Characteristics of the GRCh38 alignments. Some metrics are presented in comparison with the 1000 Genomes Project phase3 alignments to the GRCh37 assembly (numbers in parenthesis). Mapped coverage was calculated using a nominal 3 Gb genome size.

	Low coverage WGS	WES
**Sample count**	2691 (2535)	2692 (2535)
**Total bases (Gbp)**	63,744 (60,530)	28,152 (26,571)
**Total aligned bases (Gbp)**	66,437 (63,783)	30,901 (28,297)
**Percentage mapped**	96.2 (92.6)	97.5 (93.6)
**Percentage PCR duplicated**	3.6 (4.1)	11.9 (13.1)
**Mapped coverage**	8.2 (7.8)	3.8 (3.5)
**Mean target coverage**	N/A	101.09 (104.72)
**%target base 20X**	N/A	84.4 (87.24)
**CRAM file size (terabytes)**	21.2	9.3

Table 2. Comparison of GRCh37 and GRCh38 genome accessibility masks. N: bases that are "N"; L: accumulative read depth too low; H: accumulative read depth too high; Z: too many reads with mapping quality zero; Q: mapping quality less than cutoff; P: sites passed the accessibility test.

	N	L	H	Z	Q	P
**GRCh37-strict**	7.66%	1.13%	0.55%	17.20%	2.98%	70.49%
**GRCh37-pilot**	7.66%	1.13%	0.24%	2.74%		88.23%
**GRCh38-strict**	5.33%	1.44%	1.04%	18.07%	0.03%	74.09%
**GRCh38-pilot**	5.33%	1.44%	0.56%	3.67%		89.00%

Figure 1. The alignment pipeline flow chart.

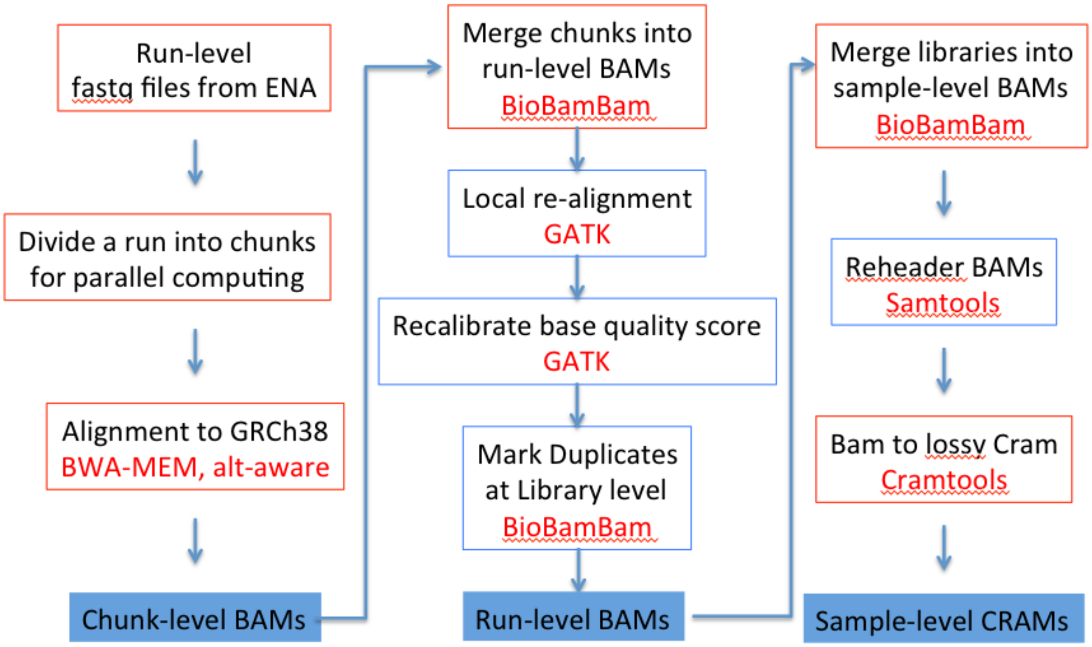

Figure 2. Measurements of mapping quality and total read depth by chromosome for the low coverage WGS sequence. A: Average mapping quality across all samples. B: Total read count per site with mapping quality of zero across all samples. C: Total read depth in all samples.

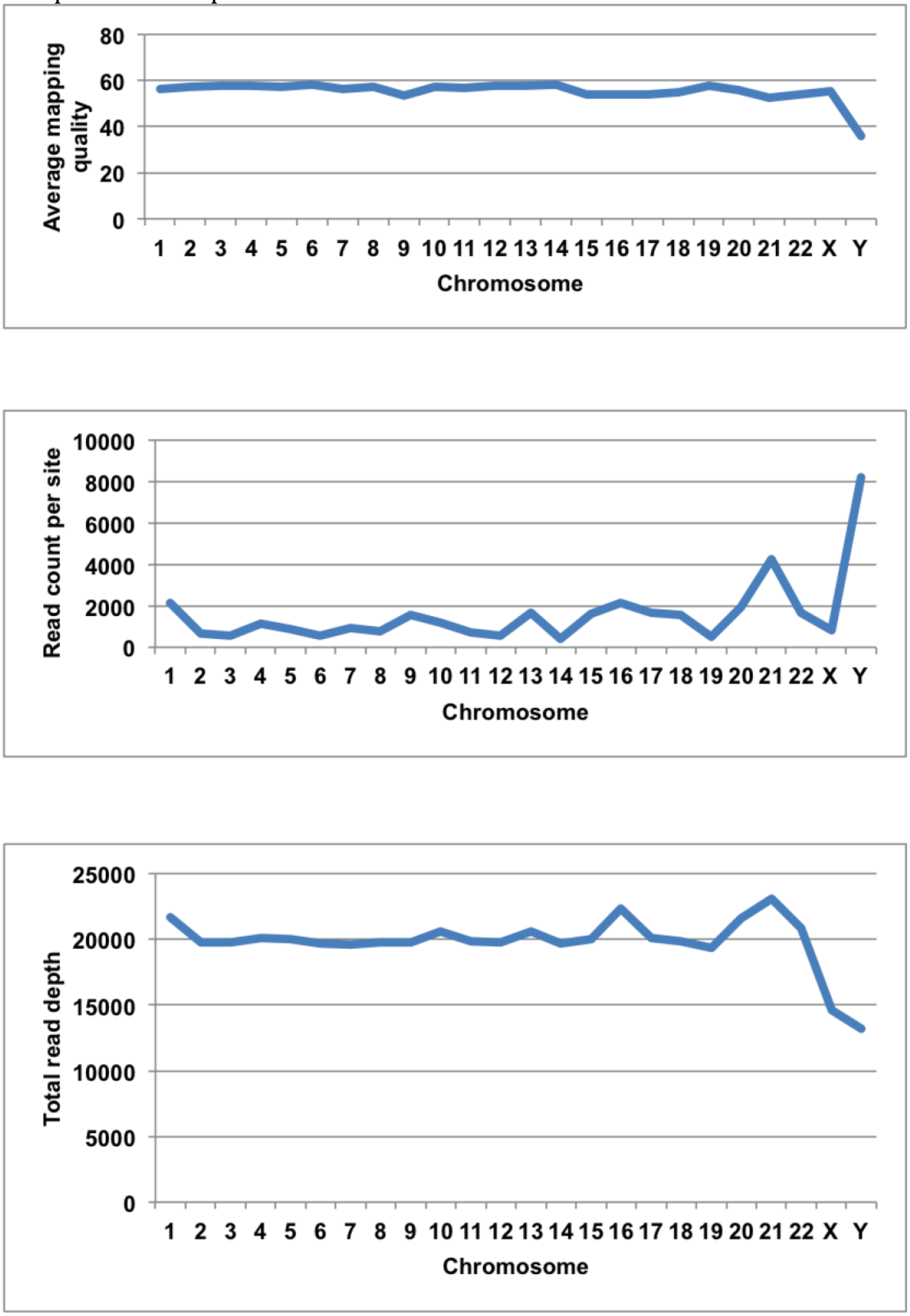

Figure 3. Percentage of sites on chromosome Y by total coverage showing the expected peak at approximately 5000x.

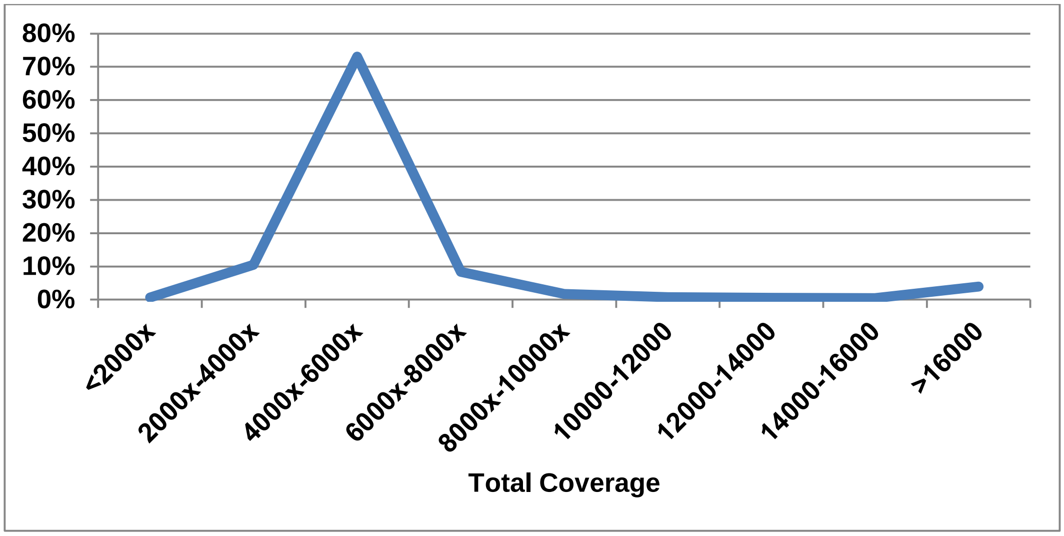

Supplemental Table 1

Click here to access/download
Supplementary Material
alignment_1kg_reads_to_GRCh38.supplementaltable1.txt

 Click here to download Personal Cover reply_to_reviewers_point_by_point_29032017a.docx

We thank the reviewers for their careful reading of the manuscript and helpful comments.

Reviewer 1:
The authors described in details how they re-aligned 1000 genome reads to the most recent human reference genome¬-GRCh38. This is not a trivial job given the large data volume. Although the workflow is similar to that used to map reads to GRCh37, it took immense computing resources to generate the resulting CRAM alignment files, and these CRAM files have great reuse potential.
Some minor concerns:
1) In Table 1, the authors should include "Total aligned bases (Gbp)" from GRCh37 for comparison.

Added; together with number of samples for GRCh37 and total bases.

2) In page 9, line53, the authors used PAR abbreviation without explanation.

Added in line and in List of Abbreviations.

3) Why Mitochondria chromosome was not included in Figure 2?

This is a technical issue caused by the software used. Figure 2 was based on numbers calculated by BamUtils, which does not produce numbers for MT.

4) In each panel of Figure 2, it would be helpful to include another line calculated from GRCh37 for side-by-side comparison.

We agree that this would be a useful comparison, but the computational requirements of calculating the numbers for the comparison are surprisingly large. Specifically, for GRCh38 BAMs, it took more than 3 weeks of time on our compute farms to get the stats for Fig 2. Each BAM was processed by BamUtils a quality matrix and then the individual matrices were merged into a matrix containing information for all BAMs. The merge step was challenging because the matrix was too big to merge all 2600 BAMs at once and so were repeatedly merged 10 matrices at a time until all merged into one.

Running the calculation for the GRCh37 BAMs would require all of the steps above plus an additional step of copying all of the BAMs into the scratch space accessible by our high memory machines. Given, this we feel that the value added to the manuscript would not be worth the effort.

5) In Figure 2C, using the median read depth (on Y-axis) across samples would be more informative and less biased by the respective sequences of the Y-chromosome.

It is true that using a median read depth in Figure 2C may bring the depth on chrY closer to expected values. However, total read depth is a standard hallmark for evaluation the outcome of any sequencing projects. It will be hard to compare to other studies if we use median coverage here. We have added a statement to the manuscript to make this point.

6) It would be helpful to other users (and the authors themselves when newer reference genome becomes available) if they could automate the whole process described in Figure 1 and make the code public available.

We have automated the process internally for use with new samples or updates to the reference genome. The goal of our analysis (and this data note) is to produce and release alignments rather than to produce and release an analysis pipeline. We would be happy to release the code using a permissive open source license (Apache v 2.0) in the interests of full transparency, but it would not run in any other system without major additional development and we do not have the capacity to either do this development ourselves or provide support for external groups starting from our code to modify it for use in another environment.

Reviewer 2:

This is a clear, well-written manuscript describing the realignment of read data from the 1000 Genomes Project to an analysis set including GRCh38, an updated version of the human reference genome assembly. This mapping task is a necessary step in the larger task of re-calling variants on the newer assembly, which the authors cite as their intention. The realignment detailed here will provide for more robust variant calling on GRCh38 and better data interpretation than variant remapping, which is compromised in regions of the assembly that have undergone change and no longer align well between versions. I support the publication of this manuscript, provided the following minor criticisms are addressed:

1. p. 3, line 35 (Bullet point #) "Substantially increase the number of alternative haplotypes associated with the assembly" The word "haplotypes" should be modified, as the Genome Reference Consortium did not require that the alternate loci scaffolds be haplotype-specific, though it is true that they increase sequence diversity in the reference (see Box 1, https://www.ncbi.nlm.nih.gov/pubmed/21750661).

Changed “hyplotypes” to “loci”.

2. p. 4, line 57 "the Epstein-Barr virus (EBV)sequence, ALT contigs, and the decoy sequences." Provide the INSDC accessions for EBV (AJ507799.2) and the decoy (GCA_000786075.2).

Added.

3. p. 4, line 56-58 "...but they are included in the reference" The word "reference" is unclear, as it may be interpreted to mean "reference assembly", rather than the alignment target reference. Suggestion: replace "reference" with "analysis set" or similar phrase.

Changed "reference" to "analysis set".

4. p. 5, line 6 "The reference data set..." Similarly, a suggestion to replace "reference" with "analysis".

Changed "The reference data set" to “The alignment target reference data set”.

5. p. 6, line Provide a URL for the NCBI Remapper. Suggested: https://www.ncbi.nlm.nih.gov/genome/tools/remap.

Added

Reviewer 3:

The manuscript describes the data not for remapping of 1000 Genome Project reads to GRCh38. Remapping to the newest reference genome is very useful for users who wish to use the resource for their own research project. Thus, the resource described in the manuscript is very precious for research community. Reviewer checked the URLs described in the manuscript and confirmed that all the URLs are alive now. Improvements (GRCh37 -> GRCh38) of mapping are successfully summarized in Table 1 and readers can easily grasp the difference.

Reviewer cannot find any information about the advantage of CRAM. Although reviewer thinks that the compression by CRAM is superior to other generic compression methods (gzip and bzip2), some real data in data size about the comparison of compression by CRAM to that by gzip and/or bzip2 might be of help to readers who wish to switch to use CRAM for the compression of their own data. It would be much more helpful if the CPU time for compression by CRAM is compared to those by gzip and/or bzip.

We believe that a comparison of compression performance of different compression methods is out of scope for this manuscript. However, we would point out that BAM is effectively gzip SAM data and, thus, the reduction from BAM to CRAM is a useful proxy for this comparison. We have added this point to the manuscript.

Minor point: Term 'HLA' is not explained in the main text while it is in the 'List of

abbreviations'.

Added

Reviewer 4:

In "Alignment of 1000 Genomes Project Reads to Reference Assembly GRCh38", Zheng-Bradley et al. describe the process and results of aligning almost 92 terabases of sequencing reads from the 1000 Genomes Project to the latest human genome reference assembly, GRCh38, including 261 alternate haplotype sequences. The magnitude of the data makes it impractical for most groups to perform these alignments on their own systems, so it is a great service to the genomics community that the authors have produced these alignments and provided them freely via the European Nucleotide Archive (ENA). The alignments will make it possible to perform variant calling on GRCh38, which will provide a more complete and robust set of variants on GRCh38 than the current set of variants remapped from GRCh37. The authors have also generated accessibility masks on GRCh38, i.e. estimates of which parts of GRCh38 can be confidently targeted by short read alignments, using the same criteria as were used on GRCh37 by the 1000 Genomes Project. I was able to find CRAM files from the project in ENA easily, and since they are provided along with .crai index files, the CRAM file URLs can be remotely accessed efficiently, e.g. as genome browser custom tracks. The manuscript is well-written. The input data and alignment process are described in detail down to the level of command lines for crucial steps. The tools used are open-source tools and the manuscript includes URLs for installing the tools. The authors provide a good comparison of the new GRCh38 alignments to the pre-existing GRCh37 alignments. I have only minor comments/questions that I would like the authors to address before publication:

Can you provide a URL for downloading the accessibility masks?

URLs are provided at the top of page 10

Methods section 4: HTSlib and Picard are mentioned without references; there should be at least a URL for each.

These have been added.

Methods section 5: It would be nice to add a brief description of the cluster used (cores, RAM etc) and the number of CPU hours. While those statistics have no bearing on the quality of the alignments, they do give a sense of the resources required for such a large endeavor.

In fact, we used different clusters over the course of the analysis, which took several

month of wall time and we did not collect metrics of CPU hours during the analysis. Moreover, we do not think the sizes of the clusters provide particularly very useful information because, even though the cluster may have thousands of nodes, each user is allowed to run only a limited number of jobs because of our fair share policy.

Technical validation section 1: regarding "The percentage of duplicated bases in the GRCh38 alignments" -- what are duplicated bases? PCR duplicates? Stretches of identical reference bases??

PCR duplication. It was mentioned explicitly in the sentence following the question ("marking PCR duplications" was done by BioBamBam).

Table 1: what is "Mapped coverage"?

Figure 3: It might be helpful to show a longer tail on the right... perhaps that might help to explain how the average coverage of Y is 13180x when it seems from Figure 3 that 90% of sites are between 2000x and 8000x. There must be some sites with extremely high coverage to pull the median of ~5000x up to an average of ~13000x.

Extended figure 3 to the right as suggested.

Usage notes (or elsewhere): how much space was saved by compressing BAM to CRAM? If it's too late to calculate because the GRCh38 BAM files are gone, at least compare to GRCh37 BAM total size. Since this is the first open CRAM dataset on thousands of genomes, it would be interesting to know how much space was saved.

Added "in our dataset, the average size for CRAMs are 28% of that of corresponding BAMs. " to page 4.

typo: "checksum is not founded in": "founded" --> "found"

Corrected

... "@SQ 'UR' field" -- briefly define "UR"

Added "which contains URI of sequences" to page 11

Availability of supporting data: "one for one analysis" -- is that "one file for one analysis"?

Changed to "one sample-level CRAM file for one analysis "

Reference typo: The authors of [10] (biobambam) are listed as "Tischler German L,

Steven." but I believe it should be "Tischler G, Leonard S".

Corrected

The first time that the terms "run" and/or "run-level" are used ("input sequence data and runs used in the alignment", "split run-level FASTQ files into chunks"), it might help to say "sequencing run" for context, for example "input sequence data and sequencing runs" and/or "split sequencing run-level FASTQ files into chunks".

Corrected as suggested.

Thanks for doing the heavy lifting -- these are definitely an improvement over GRCh37 alignments. I am very much looking forward to the freshly called variants! :)
