# Supplementary material for: Alignment of 1000 Genomes Project reads to reference assembly GRCh38
Source: Gigascience. 2017 May 20;6(7):1–8. doi: 10.1093/gigascience/gix038 (PMC5522380; doi:10.1093/gigascience/gix038)
Supplement: Response-to-Reviewer-Comments_Original-Submission.pdf [file gix038_response-to-reviewer-comments_original-submission.pdf]

# Author's Response To Reviewer Comments

Close

We thank the reviewers for their careful reading of the manuscript and helpful comments.

Reviewer 1:
The authors described in details how they re-aligned 1000 genome reads to the most recent human reference genome¬-GRCh38. This is not a trivial job given the large data volume. Although the workflow is similar to that used to map reads to GRCh37, it took immense computing resources to generate the resulting CRAM alignment files, and these CRAM files have great reuse potential.
Some minor concerns:
1) In Table 1, the authors should include "Total aligned bases (Gbp)" from GRCh37 for comparison.

Added; together with number of samples for GRCh37 and total bases.

2) In page 9, line53, the authors used PAR abbreviation without explanation.

Added in line and in List of Abbreviations.

3) Why Mitochondria chromosome was not included in Figure 2?

This is a technical issue caused by the software used. Figure 2 was based on numbers calculated by BamUtils, which does not produce numbers for MT.

4) In each panel of Figure 2, it would be helpful to include another line calculated from GRCh37 for side-by-side comparison.

We agree that this would be a useful comparison, but the computational requirements of calculating the numbers for the comparison are surprisingly large. Specifically, for GRCh38 BAMs, it took more than 3 weeks of time on our compute farms to get the stats for Fig 2. Each BAM was processed by BamUtils a quality matrix and then the individual matrices were merged into a matrix containing information for all BAMs. The merge step was challenging because the matrix was too big to merge all 2600 BAMs at once and so were repeatedly merged 10 matrices at a time until all merged into one.

Running the calculation for the GRCh37 BAMs would require all of the steps above plus an additional step of copying all of the BAMs into the scratch space accessible by our high memory machines. Given, this we feel that the value added to the manuscript would not be worth the effort.

5) In Figure 2C, using the median read depth (on Y-axis) across samples would be more informative and less biased by the respective sequences of the Y-chromosome.

It is true that using a median read depth in Figure 2C may bring the depth on chrY closer to expected values. However, total read depth is a standard hallmark for evaluation the outcome of any sequencing projects. It will be hard to compare to other studies if we use median coverage here. We have added a statement to the manuscript to make this point.

6) It would be helpful to other users (and the authors themselves when newer reference genome becomes available) if they could automate the whole process described in Figure 1 and make the code public available.

We have automated the process internally for use with new samples or updates to the reference genome. The goal of our analysis (and this data note) is to produce and release alignments rather than to produce and release an analysis pipeline. We would be happy to release the code using a permissive open source license (Apache v 2.0) in the interests of full transparency, but it would not run in any other system without major additional development and we do not have the capacity to either do this development ourselves or provide support for external groups starting from our code to modify it for use in another environment.

Reviewer 2:
This is a clear, well-written manuscript describing the realignment of read data from the 1000 Genomes Project to an analysis set including GRCh38, an updated version of the human reference genome assembly. This mapping task is a necessary step in the larger task of re-calling variants on the newer assembly, which the authors cite as their intention. The realignment detailed here will provide for more robust variant calling on GRCh38 and better data interpretation than variant remapping, which is compromised in regions of the assembly that have undergone change and no longer align well between versions. I support the publication of this manuscript, provided the following minor criticisms are addressed:
1. p. 3, line 35 (Bullet point #) "Substantially increase the number of alternative haplotypes associated with the

assembly" The word "haplotypes" should be modified, as the Genome Reference Consortium did not require that the alternate loci scaffolds be haplotype-specific, though it is true that they increase sequence diversity in the reference (see Box 1, https://www.ncbi.nlm.nih.gov/pubmed/21750661).
Changed "hyplotypes" to "loci".
2. p. 4, line 57 "the Epstein-Barr virus (EBV)sequence, ALT contigs, and the decoy sequences." Provide the INSDC accessions for EBV (AJ507799.2) and the decoy (GCA_000786075.2).
Added.
3. p. 4, line 56-58 "...but they are included in the reference" The word "reference" is unclear, as it may be interpreted to mean "reference assembly", rather than the alignment target reference. Suggestion: replace "reference" with "analysis set" or similar phrase.
Changed "reference" to "analysis set".
4. p. 5, line 6 "The reference data set..." Similarly, a suggestion to replace "reference" with "analysis".
Changed "The reference data set" to "The alignment target reference data set".
5. p. 6, line Provide a URL for the NCBI Remapper. Suggested: https://www.ncbi.nlm.nih.gov/genome/tools/remap.
Added

Reviewer 3:
The manuscript describes the data not for remapping of 1000 Genome Project reads to GRCh38. Remapping to the newest reference genome is very useful for users who wish to use the resource for their own research project. Thus, the resource described in the manuscript is very precious for research community. Reviewer checked the URLs described in the manuscript and confirmed that all the URLs are alive now. Improvements (GRCh37 -> GRCh38) of mapping are successfully summarized in Table 1 and readers can easily grasp the difference.
Reviewer cannot find any information about the advantage of CRAM. Although reviewer thinks that the compression by CRAM is superior to other generic compression methods (gzip and bzip2), some real data in data size about the comparison of compression by CRAM to that by gzip and/or bzip2 might be of help to readers who wish to switch to use CRAM for the compression of their own data. It would be much more helpful if the CPU time for compression by CRAM is compared to those by gzip and/or bzip.
We believe that a comparison of compression performance of different compression methods is out of scope for this manuscript. However, we would point out that BAM is effectively gzip SAM data and, thus, the reduction from BAM to CRAM is a useful proxy for this comparison. We have added this point to the manuscript.

Minor point: Term 'HLA' is not explained in the main text while it is in the 'List of abbreviations'.
Added

Reviewer 4:
In "Alignment of 1000 Genomes Project Reads to Reference Assembly GRCh38", Zheng-Bradley et al. describe the process and results of aligning almost 92 terabases of sequencing reads from the 1000 Genomes Project to the latest human genome reference assembly, GRCh38, including 261 alternate haplotype sequences. The magnitude of the data makes it impractical for most groups to perform these alignments on their own systems, so it is a great service to the genomics community that the authors have produced these alignments and provided them freely via the European Nucleotide Archive (ENA). The alignments will make it possible to perform variant calling on GRCh38, which will provide a more complete and robust set of variants on GRCh38 than the current set of variants remapped from GRCh37. The authors have also generated accessibility masks on GRCh38, i.e. estimates of which parts of GRCh38 can be confidently targeted by short read alignments, using the same criteria as were used on GRCh37 by the 1000 Genomes Project. I was able to find CRAM files from the project in ENA easily, and since they are provided along with .crai index files, the CRAM file URLs can be remotely accessed efficiently, e.g. as genome browser custom tracks. The manuscript is well-written. The input data and alignment process are described in detail down to the level of command lines for crucial steps. The tools used are open-source tools and the manuscript includes URLs for installing the tools. The authors provide a good comparison of the new GRCh38 alignments to the pre-existing GRCh37 alignments. I have only minor comments/questions that I would like the authors to address before publication:
Can you provide a URL for downloading the accessibility masks?
URLs are provided at the top of page 10
Methods section 4: HTSlib and Picard are mentioned without references; there should be at least a URL for each.
These have been added.
Methods section 5: It would be nice to add a brief description of the cluster used (cores, RAM etc) and the number of CPU hours. While those statistics have no bearing on the quality of the alignments, they do give a sense of the resources required for such a large endeavor.
In fact, we used different clusters over the course of the analysis, which took several month of wall time and we did not collect metrics of CPU hours during the analysis. Moreover, we do not think the sizes of the clusters provide particularly very useful information because, even though the cluster may have thousands of nodes, each user is allowed to run only a limited number of jobs because of our fair share policy.
Technical validation section 1: regarding "The percentage of duplicated bases in the GRCh38 alignments" -- what are duplicated bases? PCR duplicates? Stretches of identical reference bases??
PCR duplication. It was mentioned explicitly in the sentence following the question ("marking PCR duplications" was done by BioBamBam).
Table 1: what is "Mapped coverage"?
Figure 3: It might be helpful to show a longer tail on the right... perhaps that might help to explain how the average coverage of Y is 13180x when it seems from Figure 3 that 90% of sites are between 2000x and 8000x. There must be some sites with extremely high coverage to pull the median of ~5000x up to an average of ~13000x.
Extended figure 3 to the right as suggested.

Usage notes (or elsewhere): how much space was saved by compressing BAM to CRAM? If it's too late to calculate because the GRCh38 BAM files are gone, at least compare to GRCh37 BAM total size. Since this is the first open CRAM dataset on thousands of genomes, it would be interesting to know how much space was saved.
Added "in our dataset, the average size for CRAMs are 28% of that of corresponding BAMs. " to page 4.
typo: "checksum is not founded in": "founded" --> "found"
Corrected
... "@SQ 'UR' field" -- briefly define "UR"

Added "which contains URI of sequences" to page 11
Availability of supporting data: "one for one analysis" -- is that "one file for one analysis"?
Changed to "one sample-level CRAM file for one analysis "
Reference typo: The authors of [10] (biobambam) are listed as "Tischler German L, Steven." but I believe it should be "Tischler G, Leonard S".
Corrected
The first time that the terms "run" and/or "run-level" are used ("input sequence data and runs used in the alignment", "split run-level FASTQ files into chunks"), it might help to say "sequencing run" for context, for example "input sequence data and sequencing runs" and/or "split sequencing run-level FASTQ files into chunks".
Corrected as suggested.
Thanks for doing the heavy lifting -- these are definitely an improvement over GRCh37 alignments. I am very much looking forward to the freshly called variants! :)

Close
