# Supplementary material for: Alignment of 1000 Genomes Project reads to reference assembly GRCh38
Source: Gigascience. 2017 May 20;6(7):1–8. doi: 10.1093/gigascience/gix038 (PMC5522380; doi:10.1093/gigascience/gix038)
Supplement: Reviewer-1-Report-(Original-Submission).pdf [file gix038_reviewer-1-report-(original-submission).pdf]

Cancel Save and Close

GIGA-D-17-00028
"Alignment of 1000 Genomes Project Reads to Reference Assembly GRCh38"
Original Submission

Liquo Wang (Reviewer 1)

**Reviewer Recommendation Term:**	Minor Revision
**Rate Review:**	Please enter a number from 1-100 to rate this review (100 is best)

Custom Review Question(s)	Response
**Level of interest**<br>Please indicate how interesting you found the manuscript:	An article of importance in its field
**Quality of written English**<br>Please indicate the quality of language in the manuscript:	Acceptable
**Declaration of competing interests**<br>Please complete a declaration of competing interests, considering the following questions:<br>1. Have you in the past five years received reimbursements, fees, funding, or salary from an organisation that may in any way gain or lose financially from the publication of this manuscript, either now or in the future?<br>2. Do you hold any stocks or shares in an organisation that may in any way gain or lose financially from the publication of this manuscript, either now or in the future?<br>3. Do you hold or are you currently applying for any patents relating to the content of the manuscript?<br>4. Have you received reimbursements, fees, funding, or salary from an organization that holds or has applied for patents relating to the content of the manuscript?<br>5. Do you have any other financial competing interests?<br>6. Do you have any non-financial competing interests in relation to this paper?<br>If you can answer no to all of the above, write 'I declare that I have no competing interests' below. If your reply is yes to any, please give details below.	I declare that I have no competing interests'
I agree to the open peer review policy of the journal. I understand that my name will be included on my report to the authors and, if the manuscript is accepted for publication, my named report including any attachments I upload will be posted on the website along with the authors' responses. I agree for my report to be made available under an Open Access Creative Commons CC-BY license (http://creativecommons.org/licenses/by/4.0/). I understand that any comments which I do not wish to be included in my named report can be included as confidential comments to the editors, which will not be published.	☐ I agree to the open peer review policy of the journal
To further support our reviewers, we have joined with Publons, where you can gain additional credit to further highlight your hard work (see: https://publons.com/journal/530/gigascience). On publication of this paper, your review will be automatically	Yes

added to Publons, you can then choose whether or not to claim your Publons credit. I understand this statement.

Comments to Editor:

Comments to Author:

The authors described in details how they re-aligned 1000 genome reads to the most recent human reference genome¬-GRCh38. This is not a trivial job given the large data volume. Although the workflow is similar to that used to map reads to GRCh37, it took immense computing resources to generate the resulting CRAM alignment files, and these CRAM files have great reuse potential.

Some minor concerns:
1) In Table 1, the authors should include "Total aligned bases (Gbp)" from GRCh37 for comparison.

2) In page 9, line53, the authors used PAR abbreviation without explanation.

3) Why Mitochondria chromosome was not included in Figure 2?

4) In each panel of Figure 2, it would be helpful to include another line calculated from GRCh37 for side-by-side comparison.

5) In Figure 2C, using the median read depth (on Y-axis) across samples would be more informative and less biased by the respective sequences of the Y-chromosome.

6) It would be helpful to other users (and the authors themselves when newer reference genome becomes available) if they could automate the whole process described in Figure 1 and make the code public available.

More Reviewer Details

Cancel Save and Close
