# Supplementary material for: Alignment of 1000 Genomes Project reads to reference assembly GRCh38
Source: Gigascience. 2017 May 20;6(7):1–8. doi: 10.1093/gigascience/gix038 (PMC5522380; doi:10.1093/gigascience/gix038)
Supplement: Reviewer-2-Report-(Original-Submission).pdf [file gix038_reviewer-2-report-(original-submission).pdf]

Cancel Save and Close

GIGA-D-17-00028
"Alignment of 1000 Genomes Project Reads to Reference Assembly GRCh38"
Original Submission

Valerie Schneider (Reviewer 2)

**Reviewer Recommendation Term:**	Minor Revision
**Rate Review:**	Please enter a number from 1-100 to rate this review (100 is best)

Custom Review Question(s)	Response
**Level of interest**<br>Please indicate how interesting you found the manuscript:	An article of importance in its field
**Quality of written English**<br>Please indicate the quality of language in the manuscript:	Acceptable
**Declaration of competing interests**<br>Please complete a declaration of competing interests, considering the following questions:<br>1. Have you in the past five years received reimbursements, fees, funding, or salary from an organisation that may in any way gain or lose financially from the publication of this manuscript, either now or in the future?<br>2. Do you hold any stocks or shares in an organisation that may in any way gain or lose financially from the publication of this manuscript, either now or in the future?<br>3. Do you hold or are you currently applying for any patents relating to the content of the manuscript?<br>4. Have you received reimbursements, fees, funding, or salary from an organization that holds or has applied for patents relating to the content of the manuscript?<br>5. Do you have any other financial competing interests?<br>6. Do you have any non-financial competing interests in relation to this paper?<br>If you can answer no to all of the above, write 'I declare that I have no competing interests' below. If your reply is yes to any, please give details below.	I declare that I have no competing interests
I agree to the open peer review policy of the journal. I understand that my name will be included on my report to the authors and, if the manuscript is accepted for publication, my named report including any attachments I upload will be posted on the website along with the authors' responses. I agree for my report to be made available under an Open Access Creative Commons CC-BY license (http://creativecommons.org/licenses/by/4.0/). I understand that any comments which I do not wish to be included in my named report can be included as confidential comments to the editors, which will not be published.	☐ I agree to the open peer review policy of the journal
To further support our reviewers, we have joined with Publons, where you can gain additional credit to further highlight your hard work (see: https://publons.com/journal/530/gigascience). On publication of this paper, your review will be automatically	Yes

added to Publons, you can then choose whether or not to claim your Publons credit. I understand this statement.

Comments to Editor:

Comments to Author:

This is a clear, well-written manuscript describing the realignment of read data from the 1000 Genomes Project to an analysis set including GRCh38, an updated version of the human reference genome assembly. This mapping task is a necessary step in the larger task of re-calling variants on the newer assembly, which the authors cite as their intention. The realignment detailed here will provide for more robust variant calling on GRCh38 and better data interpretation than variant remapping, which is compromised in regions of the assembly that have undergone change and no longer align well between versions. I support the publication of this manuscript, provided the following minor criticisms are addressed:

1. p. 3, line 35 (Bullet point #) "Substantially increase the number of alternative haplotypes associated with the assembly"
The word "haplotypes" should be modified, as the Genome Reference Consortium did not require that the alternate loci scaffolds be haplotype-specific, though it is true that they increase sequence diversity in the reference (see Box 1, https://www.ncbi.nlm.nih.gov/pubmed/21750661).

2. p. 4, line 57 "the Epstein-Barr virus (EBV)sequence, ALT contigs, and the decoy sequences."
Provide the INSDC accessions for EBV (AJ507799.2) and the decoy (GCA_000786075.2).

3. p. 4, line 56-58 "...but they are included in the reference"
The word "reference" is unclear, as it may be interpreted to mean "reference assembly", rather than the alignment target reference. Suggestion: replace "reference" with "analysis set" or similar phrase.

4. p. 5, line 6 "The reference data set..."
Similarly, a suggestion to replace "reference" with "analysis".

5. p. 6, line
Provide a URL for the NCBI Remapper. Suggested: https://www.ncbi.nlm.nih.gov/genome/tools/remap.

More Reviewer Details

Cancel Save and Close
