# Supplementary material for: Alignment of 1000 Genomes Project reads to reference assembly GRCh38
Source: Gigascience. 2017 May 20;6(7):1–8. doi: 10.1093/gigascience/gix038 (PMC5522380; doi:10.1093/gigascience/gix038)
Supplement: Reviewer-3-Report-(Original-Submission).pdf [file gix038_reviewer-3-report-(original-submission).pdf]

Cancel Save and Close

GIGA-D-17-00028
"Alignment of 1000 Genomes Project Reads to Reference Assembly GRCh38"
Original Submission

Hidemasa Bono (Reviewer 3)

**Reviewer Recommendation Term:**	Minor Revision
**Rate Review:**	Please enter a number from 1-100 to rate this review (100 is best)

Custom Review Question(s)	Response
**Level of interest**<br>Please indicate how interesting you found the manuscript:	An article of importance in its field
**Quality of written English**<br>Please indicate the quality of language in the manuscript:	Acceptable
**Declaration of competing interests**<br>Please complete a declaration of competing interests, considering the following questions:<br><br>1. Have you in the past five years received reimbursements, fees, funding, or salary from an organisation that may in any way gain or lose financially from the publication of this manuscript, either now or in the future?<br>2. Do you hold any stocks or shares in an organisation that may in any way gain or lose financially from the publication of this manuscript, either now or in the future?<br>3. Do you hold or are you currently applying for any patents relating to the content of the manuscript?<br>4. Have you received reimbursements, fees, funding, or salary from an organization that holds or has applied for patents relating to the content of the manuscript?<br>5. Do you have any other financial competing interests?<br>6. Do you have any non-financial competing interests in relation to this paper?<br><br>If you can answer no to all of the above, write 'I declare that I have no competing interests' below. If your reply is yes to any, please give details below.	I declare that I have no competing interests
I agree to the open peer review policy of the journal. I understand that my name will be included on my report to the authors and, if the manuscript is accepted for publication, my named report including any attachments I upload will be posted on the website along with the authors' responses. I agree for my report to be made available under an Open Access Creative Commons CC-BY license (http://creativecommons.org/licenses/by/4.0/). I understand that any comments which I do not wish to be included in my named report can be included as confidential comments to the editors, which will not be published.	☐ I agree to the open peer review policy of the journal
To further support our reviewers, we have joined with Publons, where you can gain additional credit to further highlight your hard work (see: https://publons.com/journal/530/gigascience). On publication of this paper, your review will be automatically	Yes

added to Publons, you can then choose whether or not to claim your Publons credit. I understand this statement.

Comments to Editor:

The manuscript is very helpful to users who wish to use GRCh38 for human reference genome.
The manuscript is acceptable after a few minor revisions described above is solved.

Comments to Author:

The manuscript describes the data not for remapping of 1000 Genome Project reads to GRCh38.
Remapping to the newest reference genome is very useful for users who wish to use the resource for their own research project.
Thus, the resource described in the manuscript is very precious for research community.

Reviewer checked the URLs described in the manuscript and confirmed that all the URLs are alive now.
Improvements (GRCh37 -> GRCh38) of mapping are successfully summarized in Table 1
and readers can easily grasp the difference.

Reviewer cannot find any information about the advantage of CRAM.
Although reviewer thinks that the compression by CRAM is superior to other generic compression methods (gzip and bzip2),
some real data in data size about the comparison of compression by CRAM to that by gzip and/or bzip2 might be of help to readers
who wish to switch to use CRAM for the compression of their own data.
It would be much more helpful if the CPU time for compression by CRAM is compared to those by gzip and/or bzip.

Minor point: Term 'HLA' is not explained in the main text while it is in the 'List of abbreviations'.

More Reviewer Details

Cancel Save and Close
