# Supplementary material for: Alignment of 1000 Genomes Project reads to reference assembly GRCh38
Source: Gigascience. 2017 May 20;6(7):1–8. doi: 10.1093/gigascience/gix038 (PMC5522380; doi:10.1093/gigascience/gix038)
Supplement: Reviewer-4-Report-(Original-Submission).pdf [file gix038_reviewer-4-report-(original-submission).pdf]

Cancel Save and Close

GIGA-D-17-00028
"Alignment of 1000 Genomes Project Reads to Reference Assembly GRCh38"
Original Submission

Angie Hinrichs (Reviewer 4)

**Reviewer Recommendation Term:**	Minor Revision
**Rate Review:**	Please enter a number from 1-100 to rate this review (100 is best)

Custom Review Question(s)	Response
**Level of interest**<br>Please indicate how interesting you found the manuscript:	An article whose findings are important to those with closely related research interests
**Quality of written English**<br>Please indicate the quality of language in the manuscript:	Acceptable
**Declaration of competing interests**<br>Please complete a declaration of competing interests, considering the following questions:<br>1. Have you in the past five years received reimbursements, fees, funding, or salary from an organisation that may in any way gain or lose financially from the publication of this manuscript, either now or in the future?<br>2. Do you hold any stocks or shares in an organisation that may in any way gain or lose financially from the publication of this manuscript, either now or in the future?<br>3. Do you hold or are you currently applying for any patents relating to the content of the manuscript?<br>4. Have you received reimbursements, fees, funding, or salary from an organization that holds or has applied for patents relating to the content of the manuscript?<br>5. Do you have any other financial competing interests?<br>6. Do you have any non-financial competing interests in relation to this paper?<br>If you can answer no to all of the above, write 'I declare that I have no competing interests' below. If your reply is yes to any, please give details below.	I declare that I have no competing interests.
I agree to the open peer review policy of the journal. I understand that my name will be included on my report to the authors and, if the manuscript is accepted for publication, my named report including any attachments I upload will be posted on the website along with the authors' responses. I agree for my report to be made available under an Open Access Creative Commons CC-BY license (http://creativecommons.org/licenses/by/4.0/). I understand that any comments which I do not wish to be included in my named report can be included as confidential comments to the editors, which will not be published.	☐ I agree to the open peer review policy of the journal
To further support our reviewers, we have joined with Publons, where you can gain additional credit to further highlight your hard work (see: https://publons.com/journal/530/gigascience). On publication of this paper, your review will be automatically	Yes

added to Publons, you can then choose whether or not to claim your Publons credit. I understand this statement.

Comments to Editor:

Comments to Author:

In "Alignment of 1000 Genomes Project Reads to Reference Assembly GRCh38", Zheng-Bradley et al. describe the process and results of aligning almost 92 terabases of sequencing reads from the 1000 Genomes Project to the latest human genome reference assembly, GRCh38, including 261 alternate haplotype sequences. The magnitude of the data makes it impractical for most groups to perform these alignments on their own systems, so it is a great service to the genomics community that the authors have produced these alignments and provided them freely via the European Nucleotide Archive (ENA). The alignments will make it possible to perform variant calling on GRCh38, which will provide a more complete and robust set of variants on GRCh38 than the current set of variants remapped from GRCh37. The authors have also generated accessibility masks on GRCh38, i.e. estimates of which parts of GRCh38 can be confidently targeted by short read alignments, using the same criteria as were used on GRCh37 by the 1000 Genomes Project. I was able to find CRAM files from the project in ENA easily, and since they are provided along with .crai index files, the CRAM file URLs can be remotely accessed efficiently, e.g. as genome browser custom tracks.

The manuscript is well-written. The input data and alignment process are described in detail down to the level of command lines for crucial steps. The tools used are open-source tools and the manuscript includes URLs for installing the tools. The authors provide a good comparison of the new GRCh38 alignments to the pre-existing GRCh37 alignments.

I have only minor comments/questions that I would like the authors to address before publication:

Can you provide a URL for downloading the accessibility masks?

Methods section 4: HTSlib and Picard are mentioned without references; there should be at least a URL for each.

Methods section 5: It would be nice to add a brief description of the cluster used (cores, RAM etc) and the number of CPU hours. While those statistics have no bearing on the quality of the alignments, they do give a sense of the resources required for such a large endeavor.

Technical validation section 1: regarding "The percentage of duplicated bases in the GRCh38 alignments" -- what are duplicated bases? PCR duplicates? Stretches of identical reference bases??

Table 1: what is "Mapped coverage"?

Figure 3: It might be helpful to show a longer tail on the right... perhaps that might help to explain how the average coverage of Y is 13180x when it seems from Figure 3 that 90% of sites are between 2000x and 8000x. There must be some sites with extremely high coverage to pull the median of ~5000x up to an average of ~13000x.

Usage notes (or elsewhere): how much space was saved by compressing BAM to CRAM? If it's too late to calculate because the GRCh38 BAM files are gone, at least compare to GRCh37 BAM total size. Since this is the first open CRAM dataset on thousands of genomes, it would be interesting to know how much space was saved.

typo: "checksum is not founded in": "founded" --> "found"

... "@SQ 'UR' field" -- briefly define "UR"

Availability of supporting data: "one for one analysis" -- is that "one file for one analysis"?

Reference typo: The authors of [10] (biobambam) are listed as "Tischler German L, Steven." but I believe it should be "Tischler G, Leonard S".

The first time that the terms "run" and/or "run-level" are used ("input sequence data and runs used in the alignment", "split run-level FASTQ files into chunks"), it might help to say "sequencing run" for context, for

example "input sequence data and sequencing runs" and/or "split sequencing run-level FASTQ files into chunks".

Thanks for doing the heavy lifting -- these are definitely an improvement over GRCh37 alignments. I am very much looking forward to the freshly called variants! :)

More Reviewer Details

Cancel Save and Close
